# Consistency of the Neighbor-Net Algorithm

**DOI:** 10.1186/1748-7188-2-8

**Published:** 2007-06-28

**Authors:** David Bryant, Vincent Moulton, Andreas Spillner

**Affiliations:** 1 Department of Mathematics, University of Auckland, Private Bag 92019, Auckland, NZ; 2 School of Computing Sciences, University of East Anglia, Norwich, NR4 7TJ, UK

## Abstract

**Background:**

Neighbor-Net is a novel method for phylogenetic analysis that is currently being widely used in areas such as virology, bacteriology, and plant evolution. Given an input distance matrix, Neighbor-Net produces a phylogenetic network, a generalization of an evolutionary or phylogenetic tree which allows the graphical representation of conflicting phylogenetic signals.

**Results:**

In general, any network construction method should not depict more conflict than is found in the data, and, when the data is fitted well by a tree, the method should return a network that is close to this tree. In this paper we provide a formal proof that Neighbor-Net satisfies both of these requirements so that, in particular, Neighbor-Net is statistically consistent on circular distances.

## 1 Background

Phylogenetics is concerned with the construction and analysis of evolutionary or phylogenetic trees and networks to understand the evolution of species, populations and individuals [1]. Neighbor-Net is a phylogenetic analysis and data representation method introduced in [2]. It is loosely based on the popular Neighbor-Joining (NJ) method of Saitou and Nei [3], but with one fundamental difference: whereas NJ constructs phylogenetic trees, Neighbor-Net constructs phylogenetic networks. The method is widely used, in areas such as virology [4], bacteriology [5], plant evolution [6] and even linguistics [7].

Evolutionary processes such as hybridization between species, lateral transfer of genes, recombination within a population, and convergent evolution can all lead to evolutionary histories that are distinctly non tree-like. Moreover, even when the underlying evolution is tree-like, the presence of conflicting or ambiguous signal can make a single tree representation inappropriate. In these situations, phylogenetic network methods can be particularly useful (see e.g. [8]).

**Figure 1:**
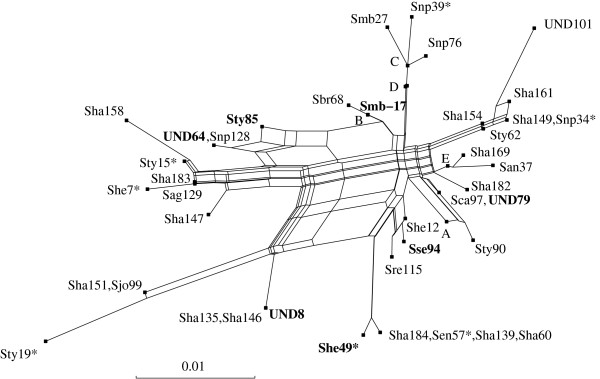
**A phylogenetic network**. The network was generated by Neighbor-Net for a sequence-based data set comprising of *Salmonella *isolates that originally appeared in [17]. A detailed network-based analysis of this data is presented in [2], where the strains indicated in bold-face are tested for the presence of recombination. Note that the network is planar (that is, it can be drawn in the plane without any crossing edges), and that parallel edges in the network represent bipartitions of the data.

Phylogenetic networks are a generalization of phylogenetic trees (see Figure 1 for a typical example of a phylogenetic network). In case there are many conflicting phylogenetic signals supported by the data, Neighbor-Net can represent this conflict graphically. In particular a single network can represent several trees simultaneously, indicate whether or not the data is substantially tree-like, and give evidence for possible reticulation or hybridization events. Evolutionary hypotheses suggested by the network can be tested directly using more detailed phylogenetic analyses and specialized biochemical methods (e.g. DNA fingerprinting or chromosome painting).

For any network construction method, it is vital that the network does not depict more conflict than is found in the data and that, if there are conflicting signals, then these should be represented by the network. At the same time, when the data is fitted well by a tree, the method should return a network that is close to being a tree. This is essential not just to avoid false inferences, but for the application of networks in statistical tests of the extent to which the data is tree-like [9].

In this paper we provide a proof that these properties all hold for Neighbor-Net. Formally, we prove that if the input to NeighborNet is a circular distance function (distance matrix) [10], then the method returns a network that exactly represents the distance. Circular distance functions are more general than additive (patristic) distances on trees and, thus, as a corollary, if Neighbor-Net is given an additive distance it will return the corresponding tree. In this sense, Neighbor-Net is a statistically consistent method.

The paper is structured as follows: In Section 2 we introduce some basic notation, and in Section 3 we review the Neighbor-Net algorithm. In Section 4 we prove that Neighbor-Net is consistent (Theorem 4.1).

## 2 Preliminaries

In this section we present some notation that will be needed to describe the Neighbor-Net algorithm. We will assume some basic facts concerning phylogenetic trees, more details concerning which may be found in [11].

Throughout this paper, *X *will denote a finite set with cardinality *n*. A *split S *= {*A*, *B*} (of *X*) is a bipartition of *X*. We let Ϭ = Ϭ(*X*) = {{*A*, *X*\*A*}|∅ ⊂ *A *⊂ *X*} denote the set of all splits of *X*, and call any non-empty subset of Ϭ(*X*) a *split system*. A *split weight function on X *is a map *ω*: Ϭ(*X*) → ℝ_≥0_. We let Ϭ_*ω *_denote the set {*S *∈ Ϭ|*ω*(*S*) > 0}, the *support *of *ω*.

Let Θ = *x*_1_, ..., *x*_*n *_be an ordering of *X*. A split *S *= {*A*, *B*} is *compatible with *Θ if there exist *i*, *j *∈ {1, ..., n}, *i *≤ *j*, such that *A *= {*x*_*i*_, ..., *x*_*j*_} or *B *= {*x*_*i*_, ..., *x*_*j*_}. Note that if a split is compatible with an ordering Θ it is also compatible with its reversal *x*_*n*_, ..., *x*_2_, *x*_1 _and with ordering *x*_2_, ..., *x*_*n*_, *x*_1_. We let Ϭ_Θ _denote the set of those splits in Ϭ(*X*) which are compatible with ordering Θ. A split system Ϭ' is *compatible with *Θ if Ϭ' ⊆ Ϭ_Θ_. In addition a split system Ϭ' ⊆ Ϭ(*X*) is *circular *if there exists an ordering Θ of *X *such that Ϭ' is compatible with Θ. Note that any split system corresponding to a phylogenetic tree is circular [[11], Ch. 3], and so circular split systems can be regarded as a generalization of split systems induced by phylogenetic trees. A split weight function *ω *is called *circular *if the split system Ϭ_*ω *_is circular. A *distance function on X *is a map *d*: *X *× *X *→ ℝ_≥0 _such that for all *x*, *y *∈ *X *both *d*(*x*, *x*) = 0 and *d*(*x*, *y*) = *d*(*y*, *x*) hold. Note that any split weight function *ω *on *X *induces a distance function *d*_*ω *_on *X *as follows: For a split *S *= {*A*, *B*} ∈ Ϭ(*X*) define the distance function or *split metric d*_*S *_by

dS(x,y)={0if {x,y}⊆A or {x,y}⊆B1otherwise,
 MathType@MTEF@5@5@+=feaafiart1ev1aaatCvAUfKttLearuWrP9MDH5MBPbIqV92AaeXatLxBI9gBaebbnrfifHhDYfgasaacH8akY=wiFfYdH8Gipec8Eeeu0xXdbba9frFj0=OqFfea0dXdd9vqai=hGuQ8kuc9pgc9s8qqaq=dirpe0xb9q8qiLsFr0=vr0=vr0dc8meaabaqaciaacaGaaeqabaqabeGadaaakeaacqWGKbazdaWgaaWcbaGaem4uamfabeaakiabcIcaOiabdIha4jabcYcaSiabdMha5jabcMcaPiabg2da9maaceqabaqbaeaabiGaaaqaaiabicdaWaqaaiabbMgaPjabbAgaMjabbccaGiabcUha7jabdIha4jabcYcaSiabdMha5jabc2ha9jabgAOinlabdgeabjabbccaGiabb+gaVjabbkhaYjabbccaGiabcUha7jabdIha4jabcYcaSiabdMha5jabc2ha9jabgAOinlabdkeacbqaaiabigdaXaqaaiabb+gaVjabbsha0jabbIgaOjabbwgaLjabbkhaYjabbEha3jabbMgaPjabbohaZjabbwgaLjabcYcaSaaaaiaawUhaaaaa@61DC@

and put

dω(x,y)=∑S∈S(X)ω(S)dS(x,y)
 MathType@MTEF@5@5@+=feaafiart1ev1aaatCvAUfKttLearuWrP9MDH5MBPbIqV92AaeXatLxBI9gBaebbnrfifHhDYfgasaacH8akY=wiFfYdH8Gipec8Eeeu0xXdbba9frFj0=OqFfea0dXdd9vqai=hGuQ8kuc9pgc9s8qqaq=dirpe0xb9q8qiLsFr0=vr0=vr0dc8meaabaqaciaacaGaaeqabaqabeGadaaakeaacqWGKbazdaWgaaWcbaacciGae8xYdChabeaakiabcIcaOiabdIha4jabcYcaSiabdMha5jabcMcaPiabg2da9maaqafabaGae8xYdCNaeiikaGIaem4uamLaeiykaKIaemizaq2aaSbaaSqaaiabdofatbqabaGccqGGOaakcqWG4baEcqGGSaalcqWG5bqEcqGGPaqkaSqaaiabdofatjabgIGioprr1ngBPrMrYf2A0vNCaeHbfv3ySLgzGyKCHTgD1jhaiqaacqGFsa=ucqGGOaakcqWGybawcqGGPaqkaeqaniabggHiLdaaaa@57D2@

for all *x*, *y *∈ *X*. A distance function *d *is called *circular *if there exits a circular split weight function *ω *such that *d *= *d*_*ω*_. An ordering Θ of *X *is said to be compatible with *d *if there exists *ω *such that *d *= *d*_*ω *_and Ϭ_*ω *_⊆ Ϭ_Θ. _Note that the representation of a circular distance function *d *is unique, i.e., if *d *= dω1
 MathType@MTEF@5@5@+=feaafiart1ev1aaatCvAUfKttLearuWrP9MDH5MBPbIqV92AaeXatLxBI9gBaebbnrfifHhDYfgasaacH8akY=wiFfYdH8Gipec8Eeeu0xXdbba9frFj0=OqFfea0dXdd9vqai=hGuQ8kuc9pgc9s8qqaq=dirpe0xb9q8qiLsFr0=vr0=vr0dc8meaabaqaciaacaGaaeqabaqabeGadaaakeaacqWGKbazdaWgaaWcbaacciGae8xYdC3aaSbaaWqaaiabigdaXaqabaaaleqaaaaa@3125@ and *d *= dω2
 MathType@MTEF@5@5@+=feaafiart1ev1aaatCvAUfKttLearuWrP9MDH5MBPbIqV92AaeXatLxBI9gBaebbnrfifHhDYfgasaacH8akY=wiFfYdH8Gipec8Eeeu0xXdbba9frFj0=OqFfea0dXdd9vqai=hGuQ8kuc9pgc9s8qqaq=dirpe0xb9q8qiLsFr0=vr0=vr0dc8meaabaqaciaacaGaaeqabaqabeGadaaakeaacqWGKbazdaWgaaWcbaacciGae8xYdC3aaSbaaWqaaiabikdaYaqabaaaleqaaaaa@3127@ for circular split weight functions *ω*_1 _and *ω*_2 _then *ω*_1 _= *ω*_2 _holds [10].

Circular distances were introduced in [10] and have been further studied in, for example, [12] and [13]. Just as any tree-like distance function on *X *can be uniquely represented by a phylogenetic tree [[11], ch. 7], any circular distance function *d *can be represented by a planar phylogenetic network such as the one pictured in Figure 1[14]. The program SplitsTree [9] allows the automatic generation of such a network for *d *by computing a circular split weight function *ω *with *d *= *d*_*ω*_.

## 3 Description of the Neighbor-Net algorithm

In this section we present a detailed description of the Neighbor-Net algorithm, as implemented in the current version of SplitsTree [9]. The Neighbor-Net algorithm was originally described in [2], where the reader may find a more informal description for how it works. For the convenience of the reader we will use the same notation as in [2] where possible.

**Figure 2:**
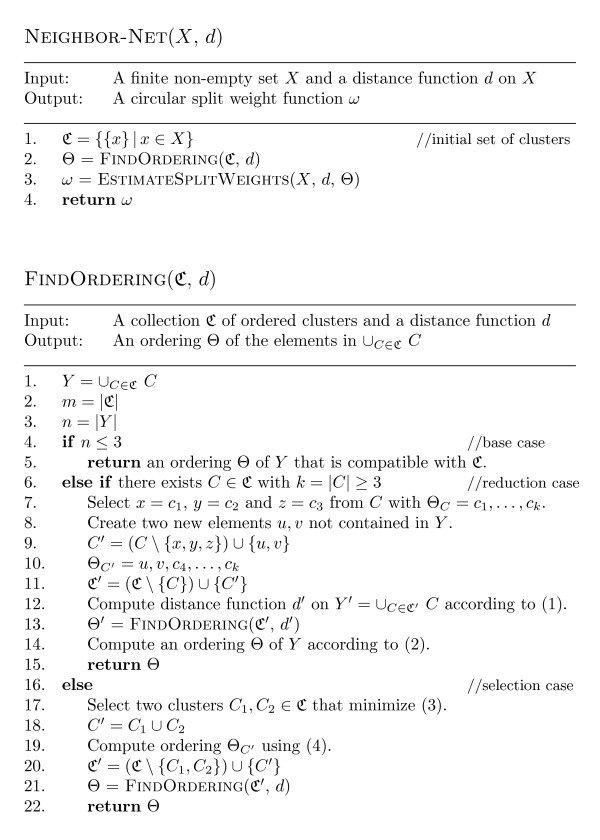
**The Neighbor-Net algorithm**. Pseudo-code for the Neighbor-Net algorithm detailing the procedure FINDORDERING.

In Figure 2 we present pseudo-code for the Neighbor-Net algorithm. The aim of the algorithm is, for a given input distance function *d*, to compute a circular split weight function *ω *so that the distance function *d*_*ω *_gives a good approximation to *d*. The resulting distance function *d*_*ω *_can then be represented by a planar phylogenetic network as indicated in the last section.

To this end, NEIGHBOR-NET first computes an ordering Θ of *X*, and then applies a non-negative least-squares procedure to find a best fit for *d *within the set of distance functions {*d*_*ϕ*_|*ϕ*:Ϭ(*X*) → ℝ_≥0_, Ϭ_*ϕ *_⊆ Ϭ_Θ_}. More details concerning the least-squares procedure may be found in [2]: Here we will concentrate on the description of the key computation for finding an ordering Θ of *X*, which is detailed in the procedure FINDORDERING.

An (*ordered*) *cluster *is a non-empty finite set *C *together with an ordering Θ_*C *_= *c*_1_, ..., *c*_*k *_of the elements in *C*, *k *= |*C*|. Two elements *a*, *b *∈ *C *are called *neighbors *if there exists *i *∈ {1, ..., *k *- 1} such that *a *= *c*_*i *_and *b *= *c*_*i*+1_, or *b *= *c*_*i *_and *a *= *c*_*i*+1_. The input of the procedure FINDORDERING consists of a set ℭ
 MathType@MTEF@5@5@+=feaafiart1ev1aaatCvAUfKttLearuWrP9MDH5MBPbIqV92AaeXatLxBI9gBaebbnrfifHhDYfgasaacH8akY=wiFfYdH8Gipec8Eeeu0xXdbba9frFj0=OqFfea0dXdd9vqai=hGuQ8kuc9pgc9s8qqaq=dirpe0xb9q8qiLsFr0=vr0=vr0dc8meaabaqaciaacaGaaeqabaqabeGadaaakeaatuuDJXwAKzKCHTgD1jharyqr1ngBPrgigjxyRrxDYbaceaGae8xlHmeaaa@3882@ of mutually disjoint clusters, together with a distance function *d *on the set Y=∪C∈ℭC
 MathType@MTEF@5@5@+=feaafiart1ev1aaatCvAUfKttLearuWrP9MDH5MBPbIqV92AaeXatLxBI9gBaebbnrfifHhDYfgasaacH8akY=wiFfYdH8Gipec8Eeeu0xXdbba9frFj0=OqFfea0dXdd9vqai=hGuQ8kuc9pgc9s8qqaq=dirpe0xb9q8qiLsFr0=vr0=vr0dc8meaabaqaciaacaGaaeqabaqabeGadaaakeaacqWGzbqwcqGH9aqpcqWIQisvdaWgaaWcbaGaem4qamKaeyicI48efv3ySLgzgjxyRrxDYbqeguuDJXwAKbIrYf2A0vNCaGabaiab=1sidbqabaGccqWGdbWqaaa@3FCD@. The ordering Θ = *y*_1_, ..., *y*_*n *_of *Y *that is returned by FINDORDERING must be *compatible *with the collection ℭ
 MathType@MTEF@5@5@+=feaafiart1ev1aaatCvAUfKttLearuWrP9MDH5MBPbIqV92AaeXatLxBI9gBaebbnrfifHhDYfgasaacH8akY=wiFfYdH8Gipec8Eeeu0xXdbba9frFj0=OqFfea0dXdd9vqai=hGuQ8kuc9pgc9s8qqaq=dirpe0xb9q8qiLsFr0=vr0=vr0dc8meaabaqaciaacaGaaeqabaqabeGadaaakeaatuuDJXwAKzKCHTgD1jharyqr1ngBPrgigjxyRrxDYbaceaGae8xlHmeaaa@3882@ of ordered clusters, that is, for every cluster *C *∈ ℭ
 MathType@MTEF@5@5@+=feaafiart1ev1aaatCvAUfKttLearuWrP9MDH5MBPbIqV92AaeXatLxBI9gBaebbnrfifHhDYfgasaacH8akY=wiFfYdH8Gipec8Eeeu0xXdbba9frFj0=OqFfea0dXdd9vqai=hGuQ8kuc9pgc9s8qqaq=dirpe0xb9q8qiLsFr0=vr0=vr0dc8meaabaqaciaacaGaaeqabaqabeGadaaakeaatuuDJXwAKzKCHTgD1jharyqr1ngBPrgigjxyRrxDYbaceaGae8xlHmeaaa@3882@ there must exist *i*, *j *∈ {1, ..., *n*}, *i *≤ *j*, with the property that Θ_*C *_= *y*_*i*_, ..., *y*_*j *_or Θ_*C *_= *y*_*j*_, ..., *y*_*i*_.

The procedure FINDORDERING calls itself recursively. Apart from the base case (line 5 of Figure 2), where the recursion bottoms out, two different cases are considered – the *reduction *and *selection *cases (lines 7–15 and lines 17–22 of Figure 2, respectively). In the reduction case a cluster *C *∈ ℭ
 MathType@MTEF@5@5@+=feaafiart1ev1aaatCvAUfKttLearuWrP9MDH5MBPbIqV92AaeXatLxBI9gBaebbnrfifHhDYfgasaacH8akY=wiFfYdH8Gipec8Eeeu0xXdbba9frFj0=OqFfea0dXdd9vqai=hGuQ8kuc9pgc9s8qqaq=dirpe0xb9q8qiLsFr0=vr0=vr0dc8meaabaqaciaacaGaaeqabaqabeGadaaakeaatuuDJXwAKzKCHTgD1jharyqr1ngBPrgigjxyRrxDYbaceaGae8xlHmeaaa@3882@ with *k *= |*C*| ≥ 3 is replaced by a smaller cluster *C*'. In particular, in lines 7–11 we let Θ_*C *_= *c*_1_, ..., *c*_*k *_be the ordering of *C *with *c*_1 _= *x*, *c*_2 _= *y*, *c*_3 _= *z*, and put *C*' = (*C*\{*x*, *y*, *z*}) ∪ {*u*, *v*} and Θ_*C*'_= *u*, *v*, *c*_4_, ..., *c*_*k*_, where *u *and *v *are two new elements not contained in *Y*. Then, in lines 12–14, we define a distance function *d*' on the set *Y*' = (*Y*\{*x*, *y*, *z*}) ∪ {*u*, *v*} using the formulae:

d′(a,b)=d(a,b)for {a,b}⊆Y′\{u,v}d′(u,a)=(α+β)d(x,a)+γd(y,a)for a∈Y′\{u,v}d′(v,a)=αd(y,a)+(β+γ)d(z,a)for a∈Y′\{u,v}d′(u,v)=αd(x,y)+βd(x,z)+γd(y,z)
 MathType@MTEF@5@5@+=feaafiart1ev1aaatCvAUfKttLearuWrP9MDH5MBPbIqV92AaeXatLxBI9gBaebbnrfifHhDYfgasaacH8akY=wiFfYdH8Gipec8Eeeu0xXdbba9frFj0=OqFfea0dXdd9vqai=hGuQ8kuc9pgc9s8qqaq=dirpe0xb9q8qiLsFr0=vr0=vr0dc8meaabaqaciaacaGaaeqabaqabeGadaaakeaafaqaaeabcaaaaeaacuWGKbazgaqbaiabcIcaOiabdggaHjabcYcaSiabdkgaIjabcMcaPiabg2da9iabdsgaKjabcIcaOiabdggaHjabcYcaSiabdkgaIjabcMcaPaqaaiabbAgaMjabb+gaVjabbkhaYjabbccaGiabcUha7jabdggaHjabcYcaSiabdkgaIjabc2ha9jabgAOinlqbdMfazzaafaGaeiixaWLaei4EaSNaemyDauNaeiilaWIaemODayNaeiyFa0habaGafmizaqMbauaacqGGOaakcqWG1bqDcqGGSaalcqWGHbqycqGGPaqkcqGH9aqpcqGGOaakiiGacqWFXoqycqGHRaWkcqWFYoGycqGGPaqkcqWGKbazcqGGOaakcqWG4baEcqGGSaalcqWGHbqycqGGPaqkcqGHRaWkcqWFZoWzcqWGKbazcqGGOaakcqWG5bqEcqGGSaalcqWGHbqycqGGPaqkaeaacqqGMbGzcqqGVbWBcqqGYbGCcqqGGaaicqWGHbqycqGHiiIZcuWGzbqwgaqbaiabcYfaCjabcUha7jabdwha1jabcYcaSiabdAha2jabc2ha9bqaaiqbdsgaKzaafaGaeiikaGIaemODayNaeiilaWIaemyyaeMaeiykaKIaeyypa0Jae8xSdeMaemizaqMaeiikaGIaemyEaKNaeiilaWIaemyyaeMaeiykaKIaey4kaSIaeiikaGIae8NSdiMaey4kaSIae83SdCMaeiykaKIaemizaqMaeiikaGIaemOEaONaeiilaWIaemyyaeMaeiykaKcabaGaeeOzayMaee4Ba8MaeeOCaiNaeeiiaaIaemyyaeMaeyicI4SafmywaKLbauaacqGGCbaxcqGG7bWEcqWG1bqDcqGGSaalcqWG2bGDcqGG9bqFaeaacuWGKbazgaqbaiabcIcaOiabdwha1jabcYcaSiabdAha2jabcMcaPiabg2da9iab=f7aHjabdsgaKjabcIcaOiabdIha4jabcYcaSiabdMha5jabcMcaPiabgUcaRiab=j7aIjabdsgaKjabcIcaOiabdIha4jabcYcaSiabdQha6jabcMcaPiabgUcaRiab=n7aNjabdsgaKjabcIcaOiabdMha5jabcYcaSiabdQha6jabcMcaPaqaaaaaaaa@D143@

where *α*, *β *and *γ *are positive real numbers satisfying *α *+ *β *+ *γ *= 1 (note that these formulae slightly differ from the ones given in [2] in which there is a typographical error). In the current implementation of Neighbor-Net the values *α *= *β *= *γ *= 1/3 are used.

When FINDORDERING is recursively called with the new collection ℭ′
 MathType@MTEF@5@5@+=feaafiart1ev1aaatCvAUfKttLearuWrP9MDH5MBPbIqV92AaeXatLxBI9gBaebbnrfifHhDYfgasaacH8akY=wiFfYdH8Gipec8Eeeu0xXdbba9frFj0=OqFfea0dXdd9vqai=hGuQ8kuc9pgc9s8qqaq=dirpe0xb9q8qiLsFr0=vr0=vr0dc8meaabaqaciaacaGaaeqabaqabeGadaaakeaatuuDJXwAKzKCHTgD1jharyqr1ngBPrgigjxyRrxDYbaceaGaf8xlHmKbauaaaaa@388E@ of clusters and distance function *d*' it returns an ordering Θ′=y′1,...,y′n−1
 MathType@MTEF@5@5@+=feaafiart1ev1aaatCvAUfKttLearuWrP9MDH5MBPbIqV92AaeXatLxBI9gBaebbnrfifHhDYfgasaacH8akY=wiFfYdH8Gipec8Eeeu0xXdbba9frFj0=OqFfea0dXdd9vqai=hGuQ8kuc9pgc9s8qqaq=dirpe0xb9q8qiLsFr0=vr0=vr0dc8meaabaqaciaacaGaaeqabaqabeGadaaakeaacuqHyoqugaqbaiabg2da9iqbdMha5zaafaWaaSbaaSqaaiabigdaXaqabaGccqGGSaalcqGGUaGlcqGGUaGlcqGGUaGlcqGGSaalcuWG5bqEgaqbamaaBaaaleaacqWGUbGBcqGHsislcqaIXaqmaeqaaaaa@3B43@ of *Y*' that is compatible with ℭ′
 MathType@MTEF@5@5@+=feaafiart1ev1aaatCvAUfKttLearuWrP9MDH5MBPbIqV92AaeXatLxBI9gBaebbnrfifHhDYfgasaacH8akY=wiFfYdH8Gipec8Eeeu0xXdbba9frFj0=OqFfea0dXdd9vqai=hGuQ8kuc9pgc9s8qqaq=dirpe0xb9q8qiLsFr0=vr0=vr0dc8meaabaqaciaacaGaaeqabaqabeGadaaakeaatuuDJXwAKzKCHTgD1jharyqr1ngBPrgigjxyRrxDYbaceaGaf8xlHmKbauaaaaa@388E@. Thus, there exists *i *∈ {1, ..., *n *- 2} such that either *u *= y′i
 MathType@MTEF@5@5@+=feaafiart1ev1aaatCvAUfKttLearuWrP9MDH5MBPbIqV92AaeXatLxBI9gBaebbnrfifHhDYfgasaacH8akY=wiFfYdH8Gipec8Eeeu0xXdbba9frFj0=OqFfea0dXdd9vqai=hGuQ8kuc9pgc9s8qqaq=dirpe0xb9q8qiLsFr0=vr0=vr0dc8meaabaqaciaacaGaaeqabaqabeGadaaakeaacuWG5bqEgaqbamaaBaaaleaacqWGPbqAaeqaaaaa@2FBA@ and *v *= y′i+1
 MathType@MTEF@5@5@+=feaafiart1ev1aaatCvAUfKttLearuWrP9MDH5MBPbIqV92AaeXatLxBI9gBaebbnrfifHhDYfgasaacH8akY=wiFfYdH8Gipec8Eeeu0xXdbba9frFj0=OqFfea0dXdd9vqai=hGuQ8kuc9pgc9s8qqaq=dirpe0xb9q8qiLsFr0=vr0=vr0dc8meaabaqaciaacaGaaeqabaqabeGadaaakeaacuWG5bqEgaqbamaaBaaaleaacqWGPbqAcqGHRaWkcqaIXaqmaeqaaaaa@318C@ or *v *= y′i
 MathType@MTEF@5@5@+=feaafiart1ev1aaatCvAUfKttLearuWrP9MDH5MBPbIqV92AaeXatLxBI9gBaebbnrfifHhDYfgasaacH8akY=wiFfYdH8Gipec8Eeeu0xXdbba9frFj0=OqFfea0dXdd9vqai=hGuQ8kuc9pgc9s8qqaq=dirpe0xb9q8qiLsFr0=vr0=vr0dc8meaabaqaciaacaGaaeqabaqabeGadaaakeaacuWG5bqEgaqbamaaBaaaleaacqWGPbqAaeqaaaaa@2FBA@ and *u *= y′i+1
 MathType@MTEF@5@5@+=feaafiart1ev1aaatCvAUfKttLearuWrP9MDH5MBPbIqV92AaeXatLxBI9gBaebbnrfifHhDYfgasaacH8akY=wiFfYdH8Gipec8Eeeu0xXdbba9frFj0=OqFfea0dXdd9vqai=hGuQ8kuc9pgc9s8qqaq=dirpe0xb9q8qiLsFr0=vr0=vr0dc8meaabaqaciaacaGaaeqabaqabeGadaaakeaacuWG5bqEgaqbamaaBaaaleaacqWGPbqAcqGHRaWkcqaIXaqmaeqaaaaa@318C@. The resulting ordering Θ of *Y *is then defined (in line 14) as follows:

Θ={y′1,...,y′i−1,x,y,z,y′i+2,...,y′n−1if u=y′i and v=y′i+1y′1,...,y′i−1,z,y,x,y′i+2,...,y′n−1if u=y′i+1 and v=y′i.
 MathType@MTEF@5@5@+=feaafiart1ev1aaatCvAUfKttLearuWrP9MDH5MBPbIqV92AaeXatLxBI9gBaebbnrfifHhDYfgasaacH8akY=wiFfYdH8Gipec8Eeeu0xXdbba9frFj0=OqFfea0dXdd9vqai=hGuQ8kuc9pgc9s8qqaq=dirpe0xb9q8qiLsFr0=vr0=vr0dc8meaabaqaciaacaGaaeqabaqabeGadaaakeaacqqHyoqucqGH9aqpdaGabeqaauaabaqaciaaaeaacuWG5bqEgaqbamaaBaaaleaacqaIXaqmaeqaaOGaeiilaWIaeiOla4IaeiOla4IaeiOla4IaeiilaWIafmyEaKNbauaadaWgaaWcbaGaemyAaKMaeyOeI0IaeGymaedabeaakiabcYcaSiabdIha4jabcYcaSiabdMha5jabcYcaSiabdQha6jabcYcaSiqbdMha5zaafaWaaSbaaSqaaiabdMgaPjabgUcaRiabikdaYaqabaGccqGGSaalcqGGUaGlcqGGUaGlcqGGUaGlcqGGSaalcuWG5bqEgaqbamaaBaaaleaacqWGUbGBcqGHsislcqaIXaqmaeqaaaGcbaGaeeyAaKMaeeOzayMaeeiiaaIaemyDauNaeyypa0JafmyEaKNbauaadaWgaaWcbaGaemyAaKgabeaakiabbccaGiabbggaHjabb6gaUjabbsgaKjabbccaGiabdAha2jabg2da9iqbdMha5zaafaWaaSbaaSqaaiabdMgaPjabgUcaRiabigdaXaqabaaakeaacuWG5bqEgaqbamaaBaaaleaacqaIXaqmaeqaaOGaeiilaWIaeiOla4IaeiOla4IaeiOla4IaeiilaWIafmyEaKNbauaadaWgaaWcbaGaemyAaKMaeyOeI0IaeGymaedabeaakiabcYcaSiabdQha6jabcYcaSiabdMha5jabcYcaSiabdIha4jabcYcaSiqbdMha5zaafaWaaSbaaSqaaiabdMgaPjabgUcaRiabikdaYaqabaGccqGGSaalcqGGUaGlcqGGUaGlcqGGUaGlcqGGSaalcuWG5bqEgaqbamaaBaaaleaacqWGUbGBcqGHsislcqaIXaqmaeqaaaGcbaGaeeyAaKMaeeOzayMaeeiiaaIaemyDauNaeyypa0JafmyEaKNbauaadaWgaaWcbaGaemyAaKMaey4kaSIaeGymaedabeaakiabbccaGiabbggaHjabb6gaUjabbsgaKjabbccaGiabdAha2jabg2da9iqbdMha5zaafaWaaSbaaSqaaiabdMgaPbqabaGccqGGUaGlaaaacaGL7baaaaa@A1C7@

This completes the description of the reduction case.

We now describe the selection case. Note that in view of line 6 this case only applies if every cluster in ℭ
 MathType@MTEF@5@5@+=feaafiart1ev1aaatCvAUfKttLearuWrP9MDH5MBPbIqV92AaeXatLxBI9gBaebbnrfifHhDYfgasaacH8akY=wiFfYdH8Gipec8Eeeu0xXdbba9frFj0=OqFfea0dXdd9vqai=hGuQ8kuc9pgc9s8qqaq=dirpe0xb9q8qiLsFr0=vr0=vr0dc8meaabaqaciaacaGaaeqabaqabeGadaaakeaatuuDJXwAKzKCHTgD1jharyqr1ngBPrgigjxyRrxDYbaceaGae8xlHmeaaa@3882@ contains at most two elements. In lines 17–18, two clusters *C*_1_, *C*_2 _∈ ℭ
 MathType@MTEF@5@5@+=feaafiart1ev1aaatCvAUfKttLearuWrP9MDH5MBPbIqV92AaeXatLxBI9gBaebbnrfifHhDYfgasaacH8akY=wiFfYdH8Gipec8Eeeu0xXdbba9frFj0=OqFfea0dXdd9vqai=hGuQ8kuc9pgc9s8qqaq=dirpe0xb9q8qiLsFr0=vr0=vr0dc8meaabaqaciaacaGaaeqabaqabeGadaaakeaatuuDJXwAKzKCHTgD1jharyqr1ngBPrgigjxyRrxDYbaceaGae8xlHmeaaa@3882@ are selected and replaced by the single cluster *C*' = *C*_1 _∪ *C*_2_. The clusters *C*_1 _and *C*_2 _are selected as follows: We define a distance function d¯
 MathType@MTEF@5@5@+=feaafiart1ev1aaatCvAUfKttLearuWrP9MDH5MBPbIqV92AaeXatLxBI9gBaebbnrfifHhDYfgasaacH8akY=wiFfYdH8Gipec8Eeeu0xXdbba9frFj0=OqFfea0dXdd9vqai=hGuQ8kuc9pgc9s8qqaq=dirpe0xb9q8qiLsFr0=vr0=vr0dc8meaabaqaciaacaGaaeqabaqabeGadaaakeaacuWGKbazgaqeaaaa@2E15@ on the set of clusters ℭ
 MathType@MTEF@5@5@+=feaafiart1ev1aaatCvAUfKttLearuWrP9MDH5MBPbIqV92AaeXatLxBI9gBaebbnrfifHhDYfgasaacH8akY=wiFfYdH8Gipec8Eeeu0xXdbba9frFj0=OqFfea0dXdd9vqai=hGuQ8kuc9pgc9s8qqaq=dirpe0xb9q8qiLsFr0=vr0=vr0dc8meaabaqaciaacaGaaeqabaqabeGadaaakeaatuuDJXwAKzKCHTgD1jharyqr1ngBPrgigjxyRrxDYbaceaGae8xlHmeaaa@3882@ by

d¯(A,B)={0if A=B1|A||B|∑a∈A∑b∈Bd(a,b)if A≠B,
 MathType@MTEF@5@5@+=feaafiart1ev1aaatCvAUfKttLearuWrP9MDH5MBPbIqV92AaeXatLxBI9gBaebbnrfifHhDYfgasaacH8akY=wiFfYdH8Gipec8Eeeu0xXdbba9frFj0=OqFfea0dXdd9vqai=hGuQ8kuc9pgc9s8qqaq=dirpe0xb9q8qiLsFr0=vr0=vr0dc8meaabaqaciaacaGaaeqabaqabeGadaaakeaacuWGKbazgaqeaiabcIcaOiabdgeabjabcYcaSiabdkeacjabcMcaPiabg2da9maaceqabaqbaeaabiGaaaqaaiabicdaWaqaaiabbMgaPjabbAgaMjabbccaGiabdgeabjabg2da9iabdkeacbqaamaalaaabaGaeGymaedabaWaaqWaaeaacqWGbbqqaiaawEa7caGLiWoadaabdaqaaiabdkeacbGaay5bSlaawIa7aaaadaaeqaqaamaaqababaGaemizaqMaeiikaGIaemyyaeMaeiilaWIaemOyaiMaeiykaKcaleaacqWGIbGycqGHiiIZcqWGcbGqaeqaniabggHiLdaaleaacqWGHbqycqGHiiIZcqWGbbqqaeqaniabggHiLdaakeaacqqGPbqAcqqGMbGzcqqGGaaicqWGbbqqcqGHGjsUcqWGcbGqcqGGSaalaaaacaGL7baaaaa@5FFF@

and select *C*_1_, *C*_2 _∈ ℭ
 MathType@MTEF@5@5@+=feaafiart1ev1aaatCvAUfKttLearuWrP9MDH5MBPbIqV92AaeXatLxBI9gBaebbnrfifHhDYfgasaacH8akY=wiFfYdH8Gipec8Eeeu0xXdbba9frFj0=OqFfea0dXdd9vqai=hGuQ8kuc9pgc9s8qqaq=dirpe0xb9q8qiLsFr0=vr0=vr0dc8meaabaqaciaacaGaaeqabaqabeGadaaakeaatuuDJXwAKzKCHTgD1jharyqr1ngBPrgigjxyRrxDYbaceaGae8xlHmeaaa@3882@, *C*_1 _≠ *C*_2 _that minimize the quantity

**Figure 3:**
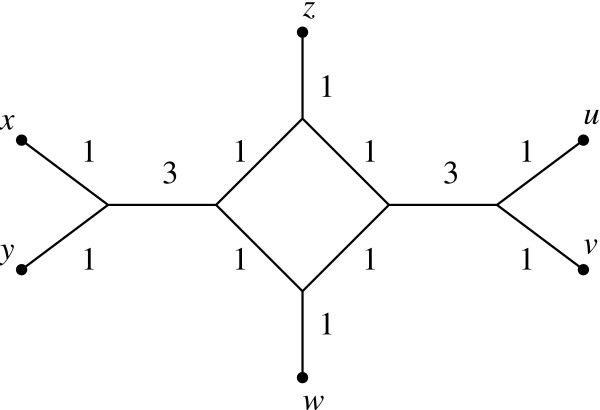
**A network representing a circular distance**. A circular distance *d *on the set {*u*, *v*, ..., *z*} for which NeighborNet using only the *Q*-criterion employed in NJ to cluster elements would be inconsistent. Distances are given by shortest paths in the network. The pairs *u*, *v *and *x*, *y *would be clustered together first and then the pair *z*, *w*. However it is not hard to show that *z *and *w *are not adjacent in any ordering of {*u*, *v*, ..., *z*} that is compatible with *d*.

Q(C1,C2)=(m−2)d¯(C1,C2)−∑C∈ℭ\{C1}d¯(C1,C)−∑C∈ℭ\{C2}d¯(C2,C)
 MathType@MTEF@5@5@+=feaafiart1ev1aaatCvAUfKttLearuWrP9MDH5MBPbIqV92AaeXatLxBI9gBaebbnrfifHhDYfgasaacH8akY=wiFfYdH8Gipec8Eeeu0xXdbba9frFj0=OqFfea0dXdd9vqai=hGuQ8kuc9pgc9s8qqaq=dirpe0xb9q8qiLsFr0=vr0=vr0dc8meaabaqaciaacaGaaeqabaqabeGadaaakeaacqWGrbqucqGGOaakcqWGdbWqdaWgaaWcbaGaeGymaedabeaakiabcYcaSiabdoeadnaaBaaaleaacqaIYaGmaeqaaOGaeiykaKIaeyypa0JaeiikaGIaemyBa0MaeyOeI0IaeGOmaiJaeiykaKIafmizaqMbaebacqGGOaakcqWGdbWqdaWgaaWcbaGaeGymaedabeaakiabcYcaSiabdoeadnaaBaaaleaacqaIYaGmaeqaaOGaeiykaKIaeyOeI0YaaabuaeaacuWGKbazgaqeaiabcIcaOiabdoeadnaaBaaaleaacqaIXaqmaeqaaOGaeiilaWIaem4qamKaeiykaKIaeyOeI0YaaabuaeaacuWGKbazgaqeaiabcIcaOiabdoeadnaaBaaaleaacqaIYaGmaeqaaOGaeiilaWIaem4qamKaeiykaKcaleaacqWGdbWqcqGHiiIZtuuDJXwAKzKCHTgD1jharyqr1ngBPrgigjxyRrxDYbaceaGae8xlHmKaeiixaWLaei4EaSNaem4qam0aaSbaaWqaaiabikdaYaqabaWccqGG9bqFaeqaniabggHiLdaaleaacqWGdbWqcqGHiiIZcqWFTeYqcqGGCbaxcqGG7bWEcqWGdbWqdaWgaaadbaGaeGymaedabeaaliabc2ha9bqab0GaeyyeIuoaaaa@76D8@

where *m *is the number of clusters in ℭ
 MathType@MTEF@5@5@+=feaafiart1ev1aaatCvAUfKttLearuWrP9MDH5MBPbIqV92AaeXatLxBI9gBaebbnrfifHhDYfgasaacH8akY=wiFfYdH8Gipec8Eeeu0xXdbba9frFj0=OqFfea0dXdd9vqai=hGuQ8kuc9pgc9s8qqaq=dirpe0xb9q8qiLsFr0=vr0=vr0dc8meaabaqaciaacaGaaeqabaqabeGadaaakeaatuuDJXwAKzKCHTgD1jharyqr1ngBPrgigjxyRrxDYbaceaGae8xlHmeaaa@3882@. The function *Q *that is used to select pairs of clusters is called the *Q-criterion*. Note that this is a direct generalization of the selection criterion used in the NJ algorithm [2]. However, using only this criterion yields a method that is not consistent as illustrated in Figure 3. So, once the clusters *C*_1 _and *C*_2 _have been selected we use a second criterion to determine an ordering Θ_*C*' _in line 19 for the new cluster *C*'. In particular, for every *x *∈ *C*_1 _∪ *C*_2 _we define

R(x)=∑C∈ℭ\{C1,C2}d¯({x},C)+∑y∈(C1∪C2)\{x}d(x,y),
 MathType@MTEF@5@5@+=feaafiart1ev1aaatCvAUfKttLearuWrP9MDH5MBPbIqV92AaeXatLxBI9gBaebbnrfifHhDYfgasaacH8akY=wiFfYdH8Gipec8Eeeu0xXdbba9frFj0=OqFfea0dXdd9vqai=hGuQ8kuc9pgc9s8qqaq=dirpe0xb9q8qiLsFr0=vr0=vr0dc8meaabaqaciaacaGaaeqabaqabeGadaaakeaacqWGsbGucqGGOaakcqWG4baEcqGGPaqkcqGH9aqpdaaeqbqaaiqbdsgaKzaaraGaeiikaGIaei4EaSNaemiEaGNaeiyFa0NaeiilaWIaem4qamKaeiykaKcaleaacqWGdbWqcqGHiiIZtuuDJXwAKzKCHTgD1jharyqr1ngBPrgigjxyRrxDYbaceaGae8xlHmKaeiixaWLaei4EaSNaem4qam0aaSbaaWqaaiabigdaXaqabaWccqGGSaalcqWGdbWqdaWgaaadbaGaeGOmaidabeaaliabc2ha9bqab0GaeyyeIuoakiabgUcaRmaaqafabaGaemizaqMaeiikaGIaemiEaGNaeiilaWIaemyEaKNaeiykaKcaleaacqWG5bqEcqGHiiIZcqGGOaakcqWGdbWqdaWgaaadbaGaeGymaedabeaaliabgQIiilabdoeadnaaBaaameaacqaIYaGmaeqaaSGaeiykaKIaeiixaWLaei4EaSNaemiEaGNaeiyFa0habeqdcqGHris5aOGaeiilaWcaaa@70F3@

put m^
 MathType@MTEF@5@5@+=feaafiart1ev1aaatCvAUfKttLearuWrP9MDH5MBPbIqV92AaeXatLxBI9gBaebbnrfifHhDYfgasaacH8akY=wiFfYdH8Gipec8Eeeu0xXdbba9frFj0=OqFfea0dXdd9vqai=hGuQ8kuc9pgc9s8qqaq=dirpe0xb9q8qiLsFr0=vr0=vr0dc8meaabaqaciaacaGaaeqabaqabeGadaaakeaacuWGTbqBgaqcaaaa@2E1F@ = *m *+ |*C*_1_| + |*C*_2_| - 2, and select *x*_1 _∈ *C*_1 _and *x*_2 _∈ *C*_2 _that minimize the quantity

Q^
 MathType@MTEF@5@5@+=feaafiart1ev1aaatCvAUfKttLearuWrP9MDH5MBPbIqV92AaeXatLxBI9gBaebbnrfifHhDYfgasaacH8akY=wiFfYdH8Gipec8Eeeu0xXdbba9frFj0=OqFfea0dXdd9vqai=hGuQ8kuc9pgc9s8qqaq=dirpe0xb9q8qiLsFr0=vr0=vr0dc8meaabaqaciaacaGaaeqabaqabeGadaaakeaacuWGrbqugaqcaaaa@2DE7@[*d*](*x*_1_, *x*_2_) = (m^
 MathType@MTEF@5@5@+=feaafiart1ev1aaatCvAUfKttLearuWrP9MDH5MBPbIqV92AaeXatLxBI9gBaebbnrfifHhDYfgasaacH8akY=wiFfYdH8Gipec8Eeeu0xXdbba9frFj0=OqFfea0dXdd9vqai=hGuQ8kuc9pgc9s8qqaq=dirpe0xb9q8qiLsFr0=vr0=vr0dc8meaabaqaciaacaGaaeqabaqabeGadaaakeaacuWGTbqBgaqcaaaa@2E1F@ - 2)*d*(*x*_1_, *x*_2_) - *R*(*x*_1_) - *R*(*x*_2_).

We then choose an ordering Θ_*C*' _in which *x*_1 _and *x*_2 _are neighbors and for which every two elements that were neighbors in *C*_1 _or *C*_2 _remain neighbors. This completes the description of the selection case, and hence the description of the procedure FINDORDERING.

## 4 Neighbor-Net is consistent

In this section we prove the consistency of Neighbor-Net:

**Theorem 4.1 **If *d*: *X *× *X *→ ℝ_≥0 _is a circular distance function, then the output of the Neighbor-Net algorithm is a circular split weight function *ω*: Ϭ(*X*) → ℝ_≥0 _with the property that *d *= *d*_*ω*_.

The key part of the Neighbor-Net algorithm is the procedure FINDORDERING. We will show that, for a circular distance function *d *= *d*_*ω *_on *X*, the call FINDORDERING({{*x*}|*x *∈ *X*}, *d*) will produce an ordering Θ of *X *that is compatible with *d*. The non-negative least squares procedure finds the distance function in {*d*_*ϕ*_|*ϕ*: Ϭ(*X*) → ℝ_≥0_, Ϭ_*ϕ *_⊆ Ϭ_Θ_} that is closest to *d*. As this set of distance functions includes *d*_*ω*_, the least squares procedure returns exactly *d *= *d*_*ω*_, proving the theorem.

We focus, then, on the proof that FINDORDERING behaves as required:

**Theorem 4.2 **Let *d*: *Y *× *Y *→ ℝ_≥0 _be a distance function that is induced by a circular split weight function *ω*: Ϭ(*Y*) → ℝ_≥0_. In addition, let ℭ
 MathType@MTEF@5@5@+=feaafiart1ev1aaatCvAUfKttLearuWrP9MDH5MBPbIqV92AaeXatLxBI9gBaebbnrfifHhDYfgasaacH8akY=wiFfYdH8Gipec8Eeeu0xXdbba9frFj0=OqFfea0dXdd9vqai=hGuQ8kuc9pgc9s8qqaq=dirpe0xb9q8qiLsFr0=vr0=vr0dc8meaabaqaciaacaGaaeqabaqabeGadaaakeaatuuDJXwAKzKCHTgD1jharyqr1ngBPrgigjxyRrxDYbaceaGae8xlHmeaaa@3882@ be a collection of mutually disjoint clusters with the property that *Y *= Y=∪C∈ℭC
 MathType@MTEF@5@5@+=feaafiart1ev1aaatCvAUfKttLearuWrP9MDH5MBPbIqV92AaeXatLxBI9gBaebbnrfifHhDYfgasaacH8akY=wiFfYdH8Gipec8Eeeu0xXdbba9frFj0=OqFfea0dXdd9vqai=hGuQ8kuc9pgc9s8qqaq=dirpe0xb9q8qiLsFr0=vr0=vr0dc8meaabaqaciaacaGaaeqabaqabeGadaaakeaacqWGzbqwcqGH9aqpcqWIQisvdaWgaaWcbaGaem4qamKaeyicI48efv3ySLgzgjxyRrxDYbqeguuDJXwAKbIrYf2A0vNCaGabaiab=1sidbqabaGccqWGdbWqaaa@3FCD@, and assume there exists an ordering of *Y *that is compatible with *ω *and with ℭ
 MathType@MTEF@5@5@+=feaafiart1ev1aaatCvAUfKttLearuWrP9MDH5MBPbIqV92AaeXatLxBI9gBaebbnrfifHhDYfgasaacH8akY=wiFfYdH8Gipec8Eeeu0xXdbba9frFj0=OqFfea0dXdd9vqai=hGuQ8kuc9pgc9s8qqaq=dirpe0xb9q8qiLsFr0=vr0=vr0dc8meaabaqaciaacaGaaeqabaqabeGadaaakeaatuuDJXwAKzKCHTgD1jharyqr1ngBPrgigjxyRrxDYbaceaGae8xlHmeaaa@3882@. Then FINDORDERING(ℭ
 MathType@MTEF@5@5@+=feaafiart1ev1aaatCvAUfKttLearuWrP9MDH5MBPbIqV92AaeXatLxBI9gBaebbnrfifHhDYfgasaacH8akY=wiFfYdH8Gipec8Eeeu0xXdbba9frFj0=OqFfea0dXdd9vqai=hGuQ8kuc9pgc9s8qqaq=dirpe0xb9q8qiLsFr0=vr0=vr0dc8meaabaqaciaacaGaaeqabaqabeGadaaakeaatuuDJXwAKzKCHTgD1jharyqr1ngBPrgigjxyRrxDYbaceaGae8xlHmeaaa@3882@, *d*) will compute an ordering that is compatible with the collection of clusters ℭ
 MathType@MTEF@5@5@+=feaafiart1ev1aaatCvAUfKttLearuWrP9MDH5MBPbIqV92AaeXatLxBI9gBaebbnrfifHhDYfgasaacH8akY=wiFfYdH8Gipec8Eeeu0xXdbba9frFj0=OqFfea0dXdd9vqai=hGuQ8kuc9pgc9s8qqaq=dirpe0xb9q8qiLsFr0=vr0=vr0dc8meaabaqaciaacaGaaeqabaqabeGadaaakeaatuuDJXwAKzKCHTgD1jharyqr1ngBPrgigjxyRrxDYbaceaGae8xlHmeaaa@3882@ and with the split weight function *ω*.

We present the proof of this result in the remainder of this section. Suppose that the algorithm FINDORDERING is called with input ℭ
 MathType@MTEF@5@5@+=feaafiart1ev1aaatCvAUfKttLearuWrP9MDH5MBPbIqV92AaeXatLxBI9gBaebbnrfifHhDYfgasaacH8akY=wiFfYdH8Gipec8Eeeu0xXdbba9frFj0=OqFfea0dXdd9vqai=hGuQ8kuc9pgc9s8qqaq=dirpe0xb9q8qiLsFr0=vr0=vr0dc8meaabaqaciaacaGaaeqabaqabeGadaaakeaatuuDJXwAKzKCHTgD1jharyqr1ngBPrgigjxyRrxDYbaceaGae8xlHmeaaa@3882@ and *d *and that there exists an ordering that is compatible with ℭ
 MathType@MTEF@5@5@+=feaafiart1ev1aaatCvAUfKttLearuWrP9MDH5MBPbIqV92AaeXatLxBI9gBaebbnrfifHhDYfgasaacH8akY=wiFfYdH8Gipec8Eeeu0xXdbba9frFj0=OqFfea0dXdd9vqai=hGuQ8kuc9pgc9s8qqaq=dirpe0xb9q8qiLsFr0=vr0=vr0dc8meaabaqaciaacaGaaeqabaqabeGadaaakeaatuuDJXwAKzKCHTgD1jharyqr1ngBPrgigjxyRrxDYbaceaGae8xlHmeaaa@3882@ and *d*. Let Y=∪C∈ℭC
 MathType@MTEF@5@5@+=feaafiart1ev1aaatCvAUfKttLearuWrP9MDH5MBPbIqV92AaeXatLxBI9gBaebbnrfifHhDYfgasaacH8akY=wiFfYdH8Gipec8Eeeu0xXdbba9frFj0=OqFfea0dXdd9vqai=hGuQ8kuc9pgc9s8qqaq=dirpe0xb9q8qiLsFr0=vr0=vr0dc8meaabaqaciaacaGaaeqabaqabeGadaaakeaacqWGzbqwcqGH9aqpcqWIQisvdaWgaaWcbaGaem4qamKaeyicI48efv3ySLgzgjxyRrxDYbqeguuDJXwAKbIrYf2A0vNCaGabaiab=1sidbqabaGccqWGdbWqaaa@3FCD@. We prove Theorem 4.2 by induction, first on |*Y*|, the cardinality of *Y*, and then on |ℭ
 MathType@MTEF@5@5@+=feaafiart1ev1aaatCvAUfKttLearuWrP9MDH5MBPbIqV92AaeXatLxBI9gBaebbnrfifHhDYfgasaacH8akY=wiFfYdH8Gipec8Eeeu0xXdbba9frFj0=OqFfea0dXdd9vqai=hGuQ8kuc9pgc9s8qqaq=dirpe0xb9q8qiLsFr0=vr0=vr0dc8meaabaqaciaacaGaaeqabaqabeGadaaakeaatuuDJXwAKzKCHTgD1jharyqr1ngBPrgigjxyRrxDYbaceaGae8xlHmeaaa@3882@|, the number of clusters in ℭ
 MathType@MTEF@5@5@+=feaafiart1ev1aaatCvAUfKttLearuWrP9MDH5MBPbIqV92AaeXatLxBI9gBaebbnrfifHhDYfgasaacH8akY=wiFfYdH8Gipec8Eeeu0xXdbba9frFj0=OqFfea0dXdd9vqai=hGuQ8kuc9pgc9s8qqaq=dirpe0xb9q8qiLsFr0=vr0=vr0dc8meaabaqaciaacaGaaeqabaqabeGadaaakeaatuuDJXwAKzKCHTgD1jharyqr1ngBPrgigjxyRrxDYbaceaGae8xlHmeaaa@3882@.

The *base case *of the induction is |*Y*| ≤ 3. In this case the set of splits Ϭ_Θ _equals Ϭ(*Y*) for every ordering of *Y*. In particular, any ordering of *Y *that is compatible with ℭ
 MathType@MTEF@5@5@+=feaafiart1ev1aaatCvAUfKttLearuWrP9MDH5MBPbIqV92AaeXatLxBI9gBaebbnrfifHhDYfgasaacH8akY=wiFfYdH8Gipec8Eeeu0xXdbba9frFj0=OqFfea0dXdd9vqai=hGuQ8kuc9pgc9s8qqaq=dirpe0xb9q8qiLsFr0=vr0=vr0dc8meaabaqaciaacaGaaeqabaqabeGadaaakeaatuuDJXwAKzKCHTgD1jharyqr1ngBPrgigjxyRrxDYbaceaGae8xlHmeaaa@3882@ is also compatible with *ω*.

We now assume that |*Y*| > 3 and make the following *induction hypothesis*:

If there exists an ordering compatible with distance function *d*' and ordered clusters ℭ′
 MathType@MTEF@5@5@+=feaafiart1ev1aaatCvAUfKttLearuWrP9MDH5MBPbIqV92AaeXatLxBI9gBaebbnrfifHhDYfgasaacH8akY=wiFfYdH8Gipec8Eeeu0xXdbba9frFj0=OqFfea0dXdd9vqai=hGuQ8kuc9pgc9s8qqaq=dirpe0xb9q8qiLsFr0=vr0=vr0dc8meaabaqaciaacaGaaeqabaqabeGadaaakeaatuuDJXwAKzKCHTgD1jharyqr1ngBPrgigjxyRrxDYbaceaGaf8xlHmKbauaaaaa@388E@, where either |∪C∈ℭ′C
 MathType@MTEF@5@5@+=feaafiart1ev1aaatCvAUfKttLearuWrP9MDH5MBPbIqV92AaeXatLxBI9gBaebbnrfifHhDYfgasaacH8akY=wiFfYdH8Gipec8Eeeu0xXdbba9frFj0=OqFfea0dXdd9vqai=hGuQ8kuc9pgc9s8qqaq=dirpe0xb9q8qiLsFr0=vr0=vr0dc8meaabaqaciaacaGaaeqabaqabeGadaaakeaacqWIQisvdaWgaaWcbaGaem4qamKaeyicI48efv3ySLgzgjxyRrxDYbqeguuDJXwAKbIrYf2A0vNCaGabaiqb=1sidzaafaaabeaakiabdoeadbaa@3D98@| < |*Y*|, or |∪C∈ℭ′C
 MathType@MTEF@5@5@+=feaafiart1ev1aaatCvAUfKttLearuWrP9MDH5MBPbIqV92AaeXatLxBI9gBaebbnrfifHhDYfgasaacH8akY=wiFfYdH8Gipec8Eeeu0xXdbba9frFj0=OqFfea0dXdd9vqai=hGuQ8kuc9pgc9s8qqaq=dirpe0xb9q8qiLsFr0=vr0=vr0dc8meaabaqaciaacaGaaeqabaqabeGadaaakeaacqWIQisvdaWgaaWcbaGaem4qamKaeyicI48efv3ySLgzgjxyRrxDYbqeguuDJXwAKbIrYf2A0vNCaGabaiqb=1sidzaafaaabeaakiabdoeadbaa@3D98@| = |*Y*| and |ℭ′
 MathType@MTEF@5@5@+=feaafiart1ev1aaatCvAUfKttLearuWrP9MDH5MBPbIqV92AaeXatLxBI9gBaebbnrfifHhDYfgasaacH8akY=wiFfYdH8Gipec8Eeeu0xXdbba9frFj0=OqFfea0dXdd9vqai=hGuQ8kuc9pgc9s8qqaq=dirpe0xb9q8qiLsFr0=vr0=vr0dc8meaabaqaciaacaGaaeqabaqabeGadaaakeaatuuDJXwAKzKCHTgD1jharyqr1ngBPrgigjxyRrxDYbaceaGaf8xlHmKbauaaaaa@388E@| < |ℭ
 MathType@MTEF@5@5@+=feaafiart1ev1aaatCvAUfKttLearuWrP9MDH5MBPbIqV92AaeXatLxBI9gBaebbnrfifHhDYfgasaacH8akY=wiFfYdH8Gipec8Eeeu0xXdbba9frFj0=OqFfea0dXdd9vqai=hGuQ8kuc9pgc9s8qqaq=dirpe0xb9q8qiLsFr0=vr0=vr0dc8meaabaqaciaacaGaaeqabaqabeGadaaakeaatuuDJXwAKzKCHTgD1jharyqr1ngBPrgigjxyRrxDYbaceaGae8xlHmeaaa@3882@|, then FINDORDERING(ℭ′
 MathType@MTEF@5@5@+=feaafiart1ev1aaatCvAUfKttLearuWrP9MDH5MBPbIqV92AaeXatLxBI9gBaebbnrfifHhDYfgasaacH8akY=wiFfYdH8Gipec8Eeeu0xXdbba9frFj0=OqFfea0dXdd9vqai=hGuQ8kuc9pgc9s8qqaq=dirpe0xb9q8qiLsFr0=vr0=vr0dc8meaabaqaciaacaGaaeqabaqabeGadaaakeaatuuDJXwAKzKCHTgD1jharyqr1ngBPrgigjxyRrxDYbaceaGaf8xlHmKbauaaaaa@388E@, *d*') will return an ordering compatible with ℭ′
 MathType@MTEF@5@5@+=feaafiart1ev1aaatCvAUfKttLearuWrP9MDH5MBPbIqV92AaeXatLxBI9gBaebbnrfifHhDYfgasaacH8akY=wiFfYdH8Gipec8Eeeu0xXdbba9frFj0=OqFfea0dXdd9vqai=hGuQ8kuc9pgc9s8qqaq=dirpe0xb9q8qiLsFr0=vr0=vr0dc8meaabaqaciaacaGaaeqabaqabeGadaaakeaatuuDJXwAKzKCHTgD1jharyqr1ngBPrgigjxyRrxDYbaceaGaf8xlHmKbauaaaaa@388E@ and *d*'.

There are two cases to consider. In the first case, ℭ
 MathType@MTEF@5@5@+=feaafiart1ev1aaatCvAUfKttLearuWrP9MDH5MBPbIqV92AaeXatLxBI9gBaebbnrfifHhDYfgasaacH8akY=wiFfYdH8Gipec8Eeeu0xXdbba9frFj0=OqFfea0dXdd9vqai=hGuQ8kuc9pgc9s8qqaq=dirpe0xb9q8qiLsFr0=vr0=vr0dc8meaabaqaciaacaGaaeqabaqabeGadaaakeaatuuDJXwAKzKCHTgD1jharyqr1ngBPrgigjxyRrxDYbaceaGae8xlHmeaaa@3882@ contains some cluster *C *with |*C*| ≥ 3. In the second case, ℭ
 MathType@MTEF@5@5@+=feaafiart1ev1aaatCvAUfKttLearuWrP9MDH5MBPbIqV92AaeXatLxBI9gBaebbnrfifHhDYfgasaacH8akY=wiFfYdH8Gipec8Eeeu0xXdbba9frFj0=OqFfea0dXdd9vqai=hGuQ8kuc9pgc9s8qqaq=dirpe0xb9q8qiLsFr0=vr0=vr0dc8meaabaqaciaacaGaaeqabaqabeGadaaakeaatuuDJXwAKzKCHTgD1jharyqr1ngBPrgigjxyRrxDYbaceaGae8xlHmeaaa@3882@ contains only clusters *C *with |*C*| ≤ 2.

### 4.1 Case 1: The reduction case

Suppose that there is *C *∈ ℭ
 MathType@MTEF@5@5@+=feaafiart1ev1aaatCvAUfKttLearuWrP9MDH5MBPbIqV92AaeXatLxBI9gBaebbnrfifHhDYfgasaacH8akY=wiFfYdH8Gipec8Eeeu0xXdbba9frFj0=OqFfea0dXdd9vqai=hGuQ8kuc9pgc9s8qqaq=dirpe0xb9q8qiLsFr0=vr0=vr0dc8meaabaqaciaacaGaaeqabaqabeGadaaakeaatuuDJXwAKzKCHTgD1jharyqr1ngBPrgigjxyRrxDYbaceaGae8xlHmeaaa@3882@ with |*C*| ≥ 3. This is the *reduction case *in the description of the algorithm. The procedure FINDORDERING constructs a new set of clusters ℭ′
 MathType@MTEF@5@5@+=feaafiart1ev1aaatCvAUfKttLearuWrP9MDH5MBPbIqV92AaeXatLxBI9gBaebbnrfifHhDYfgasaacH8akY=wiFfYdH8Gipec8Eeeu0xXdbba9frFj0=OqFfea0dXdd9vqai=hGuQ8kuc9pgc9s8qqaq=dirpe0xb9q8qiLsFr0=vr0=vr0dc8meaabaqaciaacaGaaeqabaqabeGadaaakeaatuuDJXwAKzKCHTgD1jharyqr1ngBPrgigjxyRrxDYbaceaGaf8xlHmKbauaaaaa@388E@ (in line 11) and a new distance function *d*' (in line 12). We first show that, if there is an ordering compatible with ℭ
 MathType@MTEF@5@5@+=feaafiart1ev1aaatCvAUfKttLearuWrP9MDH5MBPbIqV92AaeXatLxBI9gBaebbnrfifHhDYfgasaacH8akY=wiFfYdH8Gipec8Eeeu0xXdbba9frFj0=OqFfea0dXdd9vqai=hGuQ8kuc9pgc9s8qqaq=dirpe0xb9q8qiLsFr0=vr0=vr0dc8meaabaqaciaacaGaaeqabaqabeGadaaakeaatuuDJXwAKzKCHTgD1jharyqr1ngBPrgigjxyRrxDYbaceaGae8xlHmeaaa@3882@ and *d*, then there is also an ordering compatible with ℭ′
 MathType@MTEF@5@5@+=feaafiart1ev1aaatCvAUfKttLearuWrP9MDH5MBPbIqV92AaeXatLxBI9gBaebbnrfifHhDYfgasaacH8akY=wiFfYdH8Gipec8Eeeu0xXdbba9frFj0=OqFfea0dXdd9vqai=hGuQ8kuc9pgc9s8qqaq=dirpe0xb9q8qiLsFr0=vr0=vr0dc8meaabaqaciaacaGaaeqabaqabeGadaaakeaatuuDJXwAKzKCHTgD1jharyqr1ngBPrgigjxyRrxDYbaceaGaf8xlHmKbauaaaaa@388E@ and *d*'.

**Proposition 4.3 **If ℭ′
 MathType@MTEF@5@5@+=feaafiart1ev1aaatCvAUfKttLearuWrP9MDH5MBPbIqV92AaeXatLxBI9gBaebbnrfifHhDYfgasaacH8akY=wiFfYdH8Gipec8Eeeu0xXdbba9frFj0=OqFfea0dXdd9vqai=hGuQ8kuc9pgc9s8qqaq=dirpe0xb9q8qiLsFr0=vr0=vr0dc8meaabaqaciaacaGaaeqabaqabeGadaaakeaatuuDJXwAKzKCHTgD1jharyqr1ngBPrgigjxyRrxDYbaceaGaf8xlHmKbauaaaaa@388E@ and *d*' are constructed according to lines 7–12 of the procedure FINDORDERING then there exists an ordering compatible with ℭ′
 MathType@MTEF@5@5@+=feaafiart1ev1aaatCvAUfKttLearuWrP9MDH5MBPbIqV92AaeXatLxBI9gBaebbnrfifHhDYfgasaacH8akY=wiFfYdH8Gipec8Eeeu0xXdbba9frFj0=OqFfea0dXdd9vqai=hGuQ8kuc9pgc9s8qqaq=dirpe0xb9q8qiLsFr0=vr0=vr0dc8meaabaqaciaacaGaaeqabaqabeGadaaakeaatuuDJXwAKzKCHTgD1jharyqr1ngBPrgigjxyRrxDYbaceaGaf8xlHmKbauaaaaa@388E@ and *d*'.

*Proof*: Suppose that Θ˜
 MathType@MTEF@5@5@+=feaafiart1ev1aaatCvAUfKttLearuWrP9MDH5MBPbIqV92AaeXatLxBI9gBaebbnrfifHhDYfgasaacH8akY=wiFfYdH8Gipec8Eeeu0xXdbba9frFj0=OqFfea0dXdd9vqai=hGuQ8kuc9pgc9s8qqaq=dirpe0xb9q8qiLsFr0=vr0=vr0dc8meaabaqaciaacaGaaeqabaqabeGadaaakeaacuqHyoqugaacaaaa@2E32@ = *y*_1_, ..., *y*_*n *_is an ordering of *Y *that is compatible with ℭ
 MathType@MTEF@5@5@+=feaafiart1ev1aaatCvAUfKttLearuWrP9MDH5MBPbIqV92AaeXatLxBI9gBaebbnrfifHhDYfgasaacH8akY=wiFfYdH8Gipec8Eeeu0xXdbba9frFj0=OqFfea0dXdd9vqai=hGuQ8kuc9pgc9s8qqaq=dirpe0xb9q8qiLsFr0=vr0=vr0dc8meaabaqaciaacaGaaeqabaqabeGadaaakeaatuuDJXwAKzKCHTgD1jharyqr1ngBPrgigjxyRrxDYbaceaGae8xlHmeaaa@3882@ and *d*, where, without loss of generality, we have Θ_*C *_= *y*_1_, ..., *y*_*k*_. Let Θ˜′
 MathType@MTEF@5@5@+=feaafiart1ev1aaatCvAUfKttLearuWrP9MDH5MBPbIqV92AaeXatLxBI9gBaebbnrfifHhDYfgasaacH8akY=wiFfYdH8Gipec8Eeeu0xXdbba9frFj0=OqFfea0dXdd9vqai=hGuQ8kuc9pgc9s8qqaq=dirpe0xb9q8qiLsFr0=vr0=vr0dc8meaabaqaciaacaGaaeqabaqabeGadaaakeaacuqHyoqugaacgaqbaaaa@2E3D@ = *u*, *v*, *y*_4_, ..., *y*_*n *_= *z*_1_, ..., *z*_*n*-1_, which is an ordering of *Y*' = ∪C∈ℭ′C
 MathType@MTEF@5@5@+=feaafiart1ev1aaatCvAUfKttLearuWrP9MDH5MBPbIqV92AaeXatLxBI9gBaebbnrfifHhDYfgasaacH8akY=wiFfYdH8Gipec8Eeeu0xXdbba9frFj0=OqFfea0dXdd9vqai=hGuQ8kuc9pgc9s8qqaq=dirpe0xb9q8qiLsFr0=vr0=vr0dc8meaabaqaciaacaGaaeqabaqabeGadaaakeaacqWIQisvdaWgaaWcbaGaem4qamKaeyicI48efv3ySLgzgjxyRrxDYbqeguuDJXwAKbIrYf2A0vNCaGabaiqb=1sidzaafaaabeaakiabdoeadbaa@3D98@. We claim that the ordering Θ˜′
 MathType@MTEF@5@5@+=feaafiart1ev1aaatCvAUfKttLearuWrP9MDH5MBPbIqV92AaeXatLxBI9gBaebbnrfifHhDYfgasaacH8akY=wiFfYdH8Gipec8Eeeu0xXdbba9frFj0=OqFfea0dXdd9vqai=hGuQ8kuc9pgc9s8qqaq=dirpe0xb9q8qiLsFr0=vr0=vr0dc8meaabaqaciaacaGaaeqabaqabeGadaaakeaacuqHyoqugaacgaqbaaaa@2E3D@ is compatible with the collection ℭ′
 MathType@MTEF@5@5@+=feaafiart1ev1aaatCvAUfKttLearuWrP9MDH5MBPbIqV92AaeXatLxBI9gBaebbnrfifHhDYfgasaacH8akY=wiFfYdH8Gipec8Eeeu0xXdbba9frFj0=OqFfea0dXdd9vqai=hGuQ8kuc9pgc9s8qqaq=dirpe0xb9q8qiLsFr0=vr0=vr0dc8meaabaqaciaacaGaaeqabaqabeGadaaakeaatuuDJXwAKzKCHTgD1jharyqr1ngBPrgigjxyRrxDYbaceaGaf8xlHmKbauaaaaa@388E@ and with the distance function *d*'.

Since ℭ
 MathType@MTEF@5@5@+=feaafiart1ev1aaatCvAUfKttLearuWrP9MDH5MBPbIqV92AaeXatLxBI9gBaebbnrfifHhDYfgasaacH8akY=wiFfYdH8Gipec8Eeeu0xXdbba9frFj0=OqFfea0dXdd9vqai=hGuQ8kuc9pgc9s8qqaq=dirpe0xb9q8qiLsFr0=vr0=vr0dc8meaabaqaciaacaGaaeqabaqabeGadaaakeaatuuDJXwAKzKCHTgD1jharyqr1ngBPrgigjxyRrxDYbaceaGae8xlHmeaaa@3882@ is compatible with Θ˜
 MathType@MTEF@5@5@+=feaafiart1ev1aaatCvAUfKttLearuWrP9MDH5MBPbIqV92AaeXatLxBI9gBaebbnrfifHhDYfgasaacH8akY=wiFfYdH8Gipec8Eeeu0xXdbba9frFj0=OqFfea0dXdd9vqai=hGuQ8kuc9pgc9s8qqaq=dirpe0xb9q8qiLsFr0=vr0=vr0dc8meaabaqaciaacaGaaeqabaqabeGadaaakeaacuqHyoqugaacaaaa@2E32@ it is straight-forward to check that ℭ′
 MathType@MTEF@5@5@+=feaafiart1ev1aaatCvAUfKttLearuWrP9MDH5MBPbIqV92AaeXatLxBI9gBaebbnrfifHhDYfgasaacH8akY=wiFfYdH8Gipec8Eeeu0xXdbba9frFj0=OqFfea0dXdd9vqai=hGuQ8kuc9pgc9s8qqaq=dirpe0xb9q8qiLsFr0=vr0=vr0dc8meaabaqaciaacaGaaeqabaqabeGadaaakeaatuuDJXwAKzKCHTgD1jharyqr1ngBPrgigjxyRrxDYbaceaGaf8xlHmKbauaaaaa@388E@ is compatible with Θ˜′
 MathType@MTEF@5@5@+=feaafiart1ev1aaatCvAUfKttLearuWrP9MDH5MBPbIqV92AaeXatLxBI9gBaebbnrfifHhDYfgasaacH8akY=wiFfYdH8Gipec8Eeeu0xXdbba9frFj0=OqFfea0dXdd9vqai=hGuQ8kuc9pgc9s8qqaq=dirpe0xb9q8qiLsFr0=vr0=vr0dc8meaabaqaciaacaGaaeqabaqabeGadaaakeaacuqHyoqugaacgaqbaaaa@2E3D@. Hence, we only need to show that Θ˜′
 MathType@MTEF@5@5@+=feaafiart1ev1aaatCvAUfKttLearuWrP9MDH5MBPbIqV92AaeXatLxBI9gBaebbnrfifHhDYfgasaacH8akY=wiFfYdH8Gipec8Eeeu0xXdbba9frFj0=OqFfea0dXdd9vqai=hGuQ8kuc9pgc9s8qqaq=dirpe0xb9q8qiLsFr0=vr0=vr0dc8meaabaqaciaacaGaaeqabaqabeGadaaakeaacuqHyoqugaacgaqbaaaa@2E3D@ is compatible with *d*'. We will use a 4-point condition that was first studied in a different context by Kalmanson [15] and has been shown to characterize circular distances in [12]. To be more precise, it suffices to show that, for every four elements zi1,zi2,zi3,zi4
 MathType@MTEF@5@5@+=feaafiart1ev1aaatCvAUfKttLearuWrP9MDH5MBPbIqV92AaeXatLxBI9gBaebbnrfifHhDYfgasaacH8akY=wiFfYdH8Gipec8Eeeu0xXdbba9frFj0=OqFfea0dXdd9vqai=hGuQ8kuc9pgc9s8qqaq=dirpe0xb9q8qiLsFr0=vr0=vr0dc8meaabaqaciaacaGaaeqabaqabeGadaaakeaacqWG6bGEdaWgaaWcbaGaemyAaK2aaSbaaWqaaiabigdaXaqabaaaleqaaOGaeiilaWIaemOEaO3aaSbaaSqaaiabdMgaPnaaBaaameaacqaIYaGmaeqaaaWcbeaakiabcYcaSiabdQha6naaBaaaleaacqWGPbqAdaWgaaadbaGaeG4mamdabeaaaSqabaGccqGGSaalcqWG6bGEdaWgaaWcbaGaemyAaK2aaSbaaWqaaiabisda0aqabaaaleqaaaaa@4026@, *i*_1 _<*i*_2 _<*i*_3 _<*i*_4_,

d′(zi1,zi3)+d′(zi2,zi4)≥d′(zi1,zi2)+d′(zi3,zi4) andd′(zi1,zi3)+d′(zi2,zi4)≥d′(zi1,zi4)+d′(zi2,zi3).
 MathType@MTEF@5@5@+=feaafiart1ev1aaatCvAUfKttLearuWrP9MDH5MBPbIqV92AaeXatLxBI9gBaebbnrfifHhDYfgasaacH8akY=wiFfYdH8Gipec8Eeeu0xXdbba9frFj0=OqFfea0dXdd9vqai=hGuQ8kuc9pgc9s8qqaq=dirpe0xb9q8qiLsFr0=vr0=vr0dc8meaabaqaciaacaGaaeqabaqabeGadaaakeaafaqaaeGabaaabaGafmizaqMbauaacqGGOaakcqWG6bGEdaWgaaWcbaGaemyAaK2aaSbaaWqaaiabigdaXaqabaaaleqaaOGaeiilaWIaemOEaO3aaSbaaSqaaiabdMgaPnaaBaaameaacqaIZaWmaeqaaaWcbeaakiabcMcaPiabgUcaRiqbdsgaKzaafaGaeiikaGIaemOEaO3aaSbaaSqaaiabdMgaPnaaBaaameaacqaIYaGmaeqaaaWcbeaakiabcYcaSiabdQha6naaBaaaleaacqWGPbqAdaWgaaadbaGaeGinaqdabeaaaSqabaGccqGGPaqkcqGHLjYScuWGKbazgaqbaiabcIcaOiabdQha6naaBaaaleaacqWGPbqAdaWgaaadbaGaeGymaedabeaaaSqabaGccqGGSaalcqWG6bGEdaWgaaWcbaGaemyAaK2aaSbaaWqaaiabikdaYaqabaaaleqaaOGaeiykaKIaey4kaSIafmizaqMbauaacqGGOaakcqWG6bGEdaWgaaWcbaGaemyAaK2aaSbaaWqaaiabiodaZaqabaaaleqaaOGaeiilaWIaemOEaO3aaSbaaSqaaiabdMgaPnaaBaaameaacqaI0aanaeqaaaWcbeaakiabcMcaPiabbccaGiabbggaHjabb6gaUjabbsgaKbqaaiqbdsgaKzaafaGaeiikaGIaemOEaO3aaSbaaSqaaiabdMgaPnaaBaaameaacqaIXaqmaeqaaaWcbeaakiabcYcaSiabdQha6naaBaaaleaacqWGPbqAdaWgaaadbaGaeG4mamdabeaaaSqabaGccqGGPaqkcqGHRaWkcuWGKbazgaqbaiabcIcaOiabdQha6naaBaaaleaacqWGPbqAdaWgaaadbaGaeGOmaidabeaaaSqabaGccqGGSaalcqWG6bGEdaWgaaWcbaGaemyAaK2aaSbaaWqaaiabisda0aqabaaaleqaaOGaeiykaKIaeyyzImRafmizaqMbauaacqGGOaakcqWG6bGEdaWgaaWcbaGaemyAaK2aaSbaaWqaaiabigdaXaqabaaaleqaaOGaeiilaWIaemOEaO3aaSbaaSqaaiabdMgaPnaaBaaameaacqaI0aanaeqaaaWcbeaakiabcMcaPiabgUcaRiqbdsgaKzaafaGaeiikaGIaemOEaO3aaSbaaSqaaiabdMgaPnaaBaaameaacqaIYaGmaeqaaaWcbeaakiabcYcaSiabdQha6naaBaaaleaacqWGPbqAdaWgaaadbaGaeG4mamdabeaaaSqabaGccqGGPaqkcqGGUaGlaaaaaa@9C7A@

*Case 1*: |{zi1,zi2,zi3,zi4
 MathType@MTEF@5@5@+=feaafiart1ev1aaatCvAUfKttLearuWrP9MDH5MBPbIqV92AaeXatLxBI9gBaebbnrfifHhDYfgasaacH8akY=wiFfYdH8Gipec8Eeeu0xXdbba9frFj0=OqFfea0dXdd9vqai=hGuQ8kuc9pgc9s8qqaq=dirpe0xb9q8qiLsFr0=vr0=vr0dc8meaabaqaciaacaGaaeqabaqabeGadaaakeaacqWG6bGEdaWgaaWcbaGaemyAaK2aaSbaaWqaaiabigdaXaqabaaaleqaaOGaeiilaWIaemOEaO3aaSbaaSqaaiabdMgaPnaaBaaameaacqaIYaGmaeqaaaWcbeaakiabcYcaSiabdQha6naaBaaaleaacqWGPbqAdaWgaaadbaGaeG4mamdabeaaaSqabaGccqGGSaalcqWG6bGEdaWgaaWcbaGaemyAaK2aaSbaaWqaaiabisda0aqabaaaleqaaaaa@4026@} ∩ {*u*, *v*}| = 0. The above inequalities follow immediately since *d *is circular, and *d *and *d*' as well as Θ˜
 MathType@MTEF@5@5@+=feaafiart1ev1aaatCvAUfKttLearuWrP9MDH5MBPbIqV92AaeXatLxBI9gBaebbnrfifHhDYfgasaacH8akY=wiFfYdH8Gipec8Eeeu0xXdbba9frFj0=OqFfea0dXdd9vqai=hGuQ8kuc9pgc9s8qqaq=dirpe0xb9q8qiLsFr0=vr0=vr0dc8meaabaqaciaacaGaaeqabaqabeGadaaakeaacuqHyoqugaacaaaa@2E32@ and Θ˜′
 MathType@MTEF@5@5@+=feaafiart1ev1aaatCvAUfKttLearuWrP9MDH5MBPbIqV92AaeXatLxBI9gBaebbnrfifHhDYfgasaacH8akY=wiFfYdH8Gipec8Eeeu0xXdbba9frFj0=OqFfea0dXdd9vqai=hGuQ8kuc9pgc9s8qqaq=dirpe0xb9q8qiLsFr0=vr0=vr0dc8meaabaqaciaacaGaaeqabaqabeGadaaakeaacuqHyoqugaacgaqbaaaa@2E3D@ coincide on *Y*'\{*u*, *v*}.

*Case 2*: |{zi1,zi2,zi3,zi4
 MathType@MTEF@5@5@+=feaafiart1ev1aaatCvAUfKttLearuWrP9MDH5MBPbIqV92AaeXatLxBI9gBaebbnrfifHhDYfgasaacH8akY=wiFfYdH8Gipec8Eeeu0xXdbba9frFj0=OqFfea0dXdd9vqai=hGuQ8kuc9pgc9s8qqaq=dirpe0xb9q8qiLsFr0=vr0=vr0dc8meaabaqaciaacaGaaeqabaqabeGadaaakeaacqWG6bGEdaWgaaWcbaGaemyAaK2aaSbaaWqaaiabigdaXaqabaaaleqaaOGaeiilaWIaemOEaO3aaSbaaSqaaiabdMgaPnaaBaaameaacqaIYaGmaeqaaaWcbeaakiabcYcaSiabdQha6naaBaaaleaacqWGPbqAdaWgaaadbaGaeG4mamdabeaaaSqabaGccqGGSaalcqWG6bGEdaWgaaWcbaGaemyAaK2aaSbaaWqaaiabisda0aqabaaaleqaaaaa@4026@} ∩ {*u*, *v*}| = 1. Consider the situation zi1
 MathType@MTEF@5@5@+=feaafiart1ev1aaatCvAUfKttLearuWrP9MDH5MBPbIqV92AaeXatLxBI9gBaebbnrfifHhDYfgasaacH8akY=wiFfYdH8Gipec8Eeeu0xXdbba9frFj0=OqFfea0dXdd9vqai=hGuQ8kuc9pgc9s8qqaq=dirpe0xb9q8qiLsFr0=vr0=vr0dc8meaabaqaciaacaGaaeqabaqabeGadaaakeaacqWG6bGEdaWgaaWcbaGaemyAaK2aaSbaaWqaaiabigdaXaqabaaaleqaaaaa@30D8@ = *u*. Then

d′(zi1,zi3)+d′(zi2,zi4)=(α+β)d(x,zi3)+γd(y,zi3)+(α+β+γ)d(zi2,zi4)≥(α+β)d(x,zi2)+γd(y,zi2)+(α+β+γ)d(zi3,zi4)=d′(zi1,zi2)+d′(zi3,zi4).
 MathType@MTEF@5@5@+=feaafiart1ev1aaatCvAUfKttLearuWrP9MDH5MBPbIqV92AaeXatLxBI9gBaebbnrfifHhDYfgasaacH8akY=wiFfYdH8Gipec8Eeeu0xXdbba9frFj0=OqFfea0dXdd9vqai=hGuQ8kuc9pgc9s8qqaq=dirpe0xb9q8qiLsFr0=vr0=vr0dc8meaabaqaciaacaGaaeqabaqabeGadaaakeaafaqaaeabbaaaaeaacuWGKbazgaqbaiabcIcaOiabdQha6naaBaaaleaacqWGPbqAdaWgaaadbaGaeGymaedabeaaaSqabaGccqGGSaalcqWG6bGEdaWgaaWcbaGaemyAaK2aaSbaaWqaaiabiodaZaqabaaaleqaaOGaeiykaKIaey4kaSIafmizaqMbauaacqGGOaakcqWG6bGEdaWgaaWcbaGaemyAaK2aaSbaaWqaaiabikdaYaqabaaaleqaaOGaeiilaWIaemOEaO3aaSbaaSqaaiabdMgaPnaaBaaameaacqaI0aanaeqaaaWcbeaakiabcMcaPaqacmaa0=pa8daaefGaaCzcaiabg2da9iabcIcaOGGaciab=f7aHjabgUcaRiab=j7aIjabcMcaPiabdsgaKjabcIcaOiabdIha4jabcYcaSiabdQha6naaBaaaleaacqWGPbqAdaWgaaadbaGaeG4mamdabeaaaSqabaGccqGGPaqkcqGHRaWkcqWFZoWzcqWGKbazcqGGOaakcqWG5bqEcqGGSaalcqWG6bGEdaWgaaWcbaGaemyAaK2aaSbaaWqaaiabiodaZaqabaaaleqaaOGaeiykaKIaey4kaSIaeiikaGIae8xSdeMaey4kaSIae8NSdiMaey4kaSIae83SdCMaeiykaKIaemizaqMaeiikaGIaemOEaO3aaSbaaSqaaiabdMgaPnaaBaaameaacqaIYaGmaeqaaaWcbeaakiabcYcaSiabdQha6naaBaaaleaacqWGPbqAdaWgaaadbaGaeGinaqdabeaaaSqabaGccqGGPaqkaeGabaaibiaaxMaacqGHLjYScqGGOaakcqWFXoqycqGHRaWkcqWFYoGycqGGPaqkcqWGKbazcqGGOaakcqWG4baEcqGGSaalcqWG6bGEdaWgaaWcbaGaemyAaK2aaSbaaWqaaiabikdaYaqabaaaleqaaOGaeiykaKIaey4kaSIae83SdCMaemizaqMaeiikaGIaemyEaKNaeiilaWIaemOEaO3aaSbaaSqaaiabdMgaPnaaBaaameaacqaIYaGmaeqaaaWcbeaakiabcMcaPiabgUcaRiabcIcaOiab=f7aHjabgUcaRiab=j7aIjabgUcaRiab=n7aNjabcMcaPiabdsgaKjabcIcaOiabdQha6naaBaaaleaacqWGPbqAdaWgaaadbaGaeG4mamdabeaaaSqabaGccqGGSaalcqWG6bGEdaWgaaWcbaGaemyAaK2aaSbaaWqaaiabisda0aqabaaaleqaaOGaeiykaKcabiqaaasacaWLjaGaeyypa0JafmizaqMbauaacqGGOaakcqWG6bGEdaWgaaWcbaGaemyAaK2aaSbaaWqaaiabigdaXaqabaaaleqaaOGaeiilaWIaemOEaO3aaSbaaSqaaiabdMgaPnaaBaaameaacqaIYaGmaeqaaaWcbeaakiabcMcaPiabgUcaRiqbdsgaKzaafaGaeiikaGIaemOEaO3aaSbaaSqaaiabdMgaPnaaBaaameaacqaIZaWmaeqaaaWcbeaakiabcYcaSiabdQha6naaBaaaleaacqWGPbqAdaWgaaadbaGaeGinaqdabeaaaSqabaGccqGGPaqkcqGGUaGlaaaaaa@CC31@

The other inequalities can be derived in a completely analogous way.

*Case 3*: |{zi1,zi2,zi3,zi4
 MathType@MTEF@5@5@+=feaafiart1ev1aaatCvAUfKttLearuWrP9MDH5MBPbIqV92AaeXatLxBI9gBaebbnrfifHhDYfgasaacH8akY=wiFfYdH8Gipec8Eeeu0xXdbba9frFj0=OqFfea0dXdd9vqai=hGuQ8kuc9pgc9s8qqaq=dirpe0xb9q8qiLsFr0=vr0=vr0dc8meaabaqaciaacaGaaeqabaqabeGadaaakeaacqWG6bGEdaWgaaWcbaGaemyAaK2aaSbaaWqaaiabigdaXaqabaaaleqaaOGaeiilaWIaemOEaO3aaSbaaSqaaiabdMgaPnaaBaaameaacqaIYaGmaeqaaaWcbeaakiabcYcaSiabdQha6naaBaaaleaacqWGPbqAdaWgaaadbaGaeG4mamdabeaaaSqabaGccqGGSaalcqWG6bGEdaWgaaWcbaGaemyAaK2aaSbaaWqaaiabisda0aqabaaaleqaaaaa@4026@} ∩ {*u*, *v*}| = 2. Then we have zi1
 MathType@MTEF@5@5@+=feaafiart1ev1aaatCvAUfKttLearuWrP9MDH5MBPbIqV92AaeXatLxBI9gBaebbnrfifHhDYfgasaacH8akY=wiFfYdH8Gipec8Eeeu0xXdbba9frFj0=OqFfea0dXdd9vqai=hGuQ8kuc9pgc9s8qqaq=dirpe0xb9q8qiLsFr0=vr0=vr0dc8meaabaqaciaacaGaaeqabaqabeGadaaakeaacqWG6bGEdaWgaaWcbaGaemyAaK2aaSbaaWqaaiabigdaXaqabaaaleqaaaaa@30D8@ = *u *and zi2
 MathType@MTEF@5@5@+=feaafiart1ev1aaatCvAUfKttLearuWrP9MDH5MBPbIqV92AaeXatLxBI9gBaebbnrfifHhDYfgasaacH8akY=wiFfYdH8Gipec8Eeeu0xXdbba9frFj0=OqFfea0dXdd9vqai=hGuQ8kuc9pgc9s8qqaq=dirpe0xb9q8qiLsFr0=vr0=vr0dc8meaabaqaciaacaGaaeqabaqabeGadaaakeaacqWG6bGEdaWgaaWcbaGaemyAaK2aaSbaaWqaaiabikdaYaqabaaaleqaaaaa@30DA@ = *v *and

d′(zi1,zi3)+d′(zi2,zi4)=(α+β)d(x,zi3)+γd(y,zi3)+αd(y,zi4)+(β+γ)d(z,zi4)≥αd(x,y)+βd(x,z)+γd(y,z)+(α+β+γ)d(zi3,zi4)=d′(zi1,zi2)+d′(zi3,zi4).
 MathType@MTEF@5@5@+=feaafiart1ev1aaatCvAUfKttLearuWrP9MDH5MBPbIqV92AaeXatLxBI9gBaebbnrfifHhDYfgasaacH8akY=wiFfYdH8Gipec8Eeeu0xXdbba9frFj0=OqFfea0dXdd9vqai=hGuQ8kuc9pgc9s8qqaq=dirpe0xb9q8qiLsFr0=vr0=vr0dc8meaabaqaciaacaGaaeqabaqabeGadaaakeGacaa5aaaubuaabaqaeeaaaaqaaiqbdsgaKzaafaGaeiikaGIaemOEaO3aaSbaaSqaaiabdMgaPnaaBaaameaacqaIXaqmaeqaaaWcbeaakiabcYcaSiabdQha6naaBaaaleaacqWGPbqAdaWgaaadbaGaeG4mamdabeaaaSqabaGccqGGPaqkcqGHRaWkcuWGKbazgaqbaiabcIcaOiabdQha6naaBaaaleaacqWGPbqAdaWgaaadbaGaeGOmaidabeaaaSqabaGccqGGSaalcqWG6bGEdaWgaaWcbaGaemyAaK2aaSbaaWqaaiabisda0aqabaaaleqaaOGaeiykaKcabiqaaasacaWLjaGaeyypa0JaeiikaGccciGae8xSdeMaey4kaSIae8NSdiMaeiykaKIaemizaqMaeiikaGIaemiEaGNaeiilaWIaemOEaO3aaSbaaSqaaiabdMgaPnaaBaaameaacqaIZaWmaeqaaaWcbeaakiabcMcaPiabgUcaRiab=n7aNjabdsgaKjabcIcaOiabdMha5jabcYcaSiabdQha6naaBaaaleaacqWGPbqAdaWgaaadbaGaeG4mamdabeaaaSqabaGccqGGPaqkcqGHRaWkcqWFXoqycqWGKbazcqGGOaakcqWG5bqEcqGGSaalcqWG6bGEdaWgaaWcbaGaemyAaK2aaSbaaWqaaiabisda0aqabaaaleqaaOGaeiykaKIaey4kaSIaeiikaGIae8NSdiMaey4kaSIae83SdCMaeiykaKIaemizaqMaeiikaGIaemOEaONaeiilaWIaemOEaO3aaSbaaSqaaiabdMgaPnaaBaaameaacqaI0aanaeqaaaWcbeaakiabcMcaPaqaciaaGeaaOrGaaCzcaiabgwMiZkab=f7aHjabdsgaKjabcIcaOiabdIha4jabcYcaSiabdMha5jabcMcaPiabgUcaRiab=j7aIjabdsgaKjabcIcaOiabdIha4jabcYcaSiabdQha6jabcMcaPiabgUcaRiab=n7aNjabdsgaKjabcIcaOiabdMha5jabcYcaSiabdQha6jabcMcaPiabgUcaRiabcIcaOiab=f7aHjabgUcaRiab=j7aIjabgUcaRiab=n7aNjabcMcaPiabdsgaKjabcIcaOiabdQha6naaBaaaleaacqWGPbqAdaWgaaadbaGaeG4mamdabeaaaSqabaGccqGGSaalcqWG6bGEdaWgaaWcbaGaemyAaK2aaSbaaWqaaiabisda0aqabaaaleqaaOGaeiykaKcabiqaaGqacaWLjaGaeyypa0JafmizaqMbauaacqGGOaakcqWG6bGEdaWgaaWcbaGaemyAaK2aaSbaaWqaaiabigdaXaqabaaaleqaaOGaeiilaWIaemOEaO3aaSbaaSqaaiabdMgaPnaaBaaameaacqaIYaGmaeqaaaWcbeaakiabcMcaPiabgUcaRiqbdsgaKzaafaGaeiikaGIaemOEaO3aaSbaaSqaaiabdMgaPnaaBaaameaacqaIZaWmaeqaaaWcbeaakiabcYcaSiabdQha6naaBaaaleaacqWGPbqAdaWgaaadbaGaeGinaqdabeaaaSqabaGccqGGPaqkcqGGUaGlaaaaaa@D138@

The other inequality d′(zi1,zi3)+d′(zi2,zi4)≥d′(zi1,zi4)+d′(zi2,zi3)
 MathType@MTEF@5@5@+=feaafiart1ev1aaatCvAUfKttLearuWrP9MDH5MBPbIqV92AaeXatLxBI9gBaebbnrfifHhDYfgasaacH8akY=wiFfYdH8Gipec8Eeeu0xXdbba9frFj0=OqFfea0dXdd9vqai=hGuQ8kuc9pgc9s8qqaq=dirpe0xb9q8qiLsFr0=vr0=vr0dc8meaabaqaciaacaGaaeqabaqabeGadaaakeaacuWGKbazgaqbaiabcIcaOiabdQha6naaBaaaleaacqWGPbqAdaWgaaadbaGaeGymaedabeaaaSqabaGccqGGSaalcqWG6bGEdaWgaaWcbaGaemyAaK2aaSbaaWqaaiabiodaZaqabaaaleqaaOGaeiykaKIaey4kaSIafmizaqMbauaacqGGOaakcqWG6bGEdaWgaaWcbaGaemyAaK2aaSbaaWqaaiabikdaYaqabaaaleqaaOGaeiilaWIaemOEaO3aaSbaaSqaaiabdMgaPnaaBaaameaacqaI0aanaeqaaaWcbeaakiabcMcaPiabgwMiZkqbdsgaKzaafaGaeiikaGIaemOEaO3aaSbaaSqaaiabdMgaPnaaBaaameaacqaIXaqmaeqaaaWcbeaakiabcYcaSiabdQha6naaBaaaleaacqWGPbqAdaWgaaadbaGaeGinaqdabeaaaSqabaGccqGGPaqkcqGHRaWkcuWGKbazgaqbaiabcIcaOiabdQha6naaBaaaleaacqWGPbqAdaWgaaadbaGaeGOmaidabeaaaSqabaGccqGGSaalcqWG6bGEdaWgaaWcbaGaemyAaK2aaSbaaWqaaiabiodaZaqabaaaleqaaOGaeiykaKcaaa@61BA@ can be shown to hold in a similar way.   ■

The procedure FINDORDERING calls itself recursively with ℭ′
 MathType@MTEF@5@5@+=feaafiart1ev1aaatCvAUfKttLearuWrP9MDH5MBPbIqV92AaeXatLxBI9gBaebbnrfifHhDYfgasaacH8akY=wiFfYdH8Gipec8Eeeu0xXdbba9frFj0=OqFfea0dXdd9vqai=hGuQ8kuc9pgc9s8qqaq=dirpe0xb9q8qiLsFr0=vr0=vr0dc8meaabaqaciaacaGaaeqabaqabeGadaaakeaatuuDJXwAKzKCHTgD1jharyqr1ngBPrgigjxyRrxDYbaceaGaf8xlHmKbauaaaaa@388E@ and *d*' as input. An ordering of *Y*', the union of ℭ′
 MathType@MTEF@5@5@+=feaafiart1ev1aaatCvAUfKttLearuWrP9MDH5MBPbIqV92AaeXatLxBI9gBaebbnrfifHhDYfgasaacH8akY=wiFfYdH8Gipec8Eeeu0xXdbba9frFj0=OqFfea0dXdd9vqai=hGuQ8kuc9pgc9s8qqaq=dirpe0xb9q8qiLsFr0=vr0=vr0dc8meaabaqaciaacaGaaeqabaqabeGadaaakeaatuuDJXwAKzKCHTgD1jharyqr1ngBPrgigjxyRrxDYbaceaGaf8xlHmKbauaaaaa@388E@, is returned. By Proposition 4.3 and the induction hypothesis, this ordering Θ' is compatible with ℭ′
 MathType@MTEF@5@5@+=feaafiart1ev1aaatCvAUfKttLearuWrP9MDH5MBPbIqV92AaeXatLxBI9gBaebbnrfifHhDYfgasaacH8akY=wiFfYdH8Gipec8Eeeu0xXdbba9frFj0=OqFfea0dXdd9vqai=hGuQ8kuc9pgc9s8qqaq=dirpe0xb9q8qiLsFr0=vr0=vr0dc8meaabaqaciaacaGaaeqabaqabeGadaaakeaatuuDJXwAKzKCHTgD1jharyqr1ngBPrgigjxyRrxDYbaceaGaf8xlHmKbauaaaaa@388E@ and *d*'. It is used to construct an ordering Θ on *Y*, in line 14, which becomes the output of the procedure.

**Proposition 4.4 **The ordering Θ is compatible with collection ℭ
 MathType@MTEF@5@5@+=feaafiart1ev1aaatCvAUfKttLearuWrP9MDH5MBPbIqV92AaeXatLxBI9gBaebbnrfifHhDYfgasaacH8akY=wiFfYdH8Gipec8Eeeu0xXdbba9frFj0=OqFfea0dXdd9vqai=hGuQ8kuc9pgc9s8qqaq=dirpe0xb9q8qiLsFr0=vr0=vr0dc8meaabaqaciaacaGaaeqabaqabeGadaaakeaatuuDJXwAKzKCHTgD1jharyqr1ngBPrgigjxyRrxDYbaceaGae8xlHmeaaa@3882@ and with the distance function *d*.

*Proof*: Since ℭ′
 MathType@MTEF@5@5@+=feaafiart1ev1aaatCvAUfKttLearuWrP9MDH5MBPbIqV92AaeXatLxBI9gBaebbnrfifHhDYfgasaacH8akY=wiFfYdH8Gipec8Eeeu0xXdbba9frFj0=OqFfea0dXdd9vqai=hGuQ8kuc9pgc9s8qqaq=dirpe0xb9q8qiLsFr0=vr0=vr0dc8meaabaqaciaacaGaaeqabaqabeGadaaakeaatuuDJXwAKzKCHTgD1jharyqr1ngBPrgigjxyRrxDYbaceaGaf8xlHmKbauaaaaa@388E@ is compatible with Θ' it is straight-forward to check that ℭ
 MathType@MTEF@5@5@+=feaafiart1ev1aaatCvAUfKttLearuWrP9MDH5MBPbIqV92AaeXatLxBI9gBaebbnrfifHhDYfgasaacH8akY=wiFfYdH8Gipec8Eeeu0xXdbba9frFj0=OqFfea0dXdd9vqai=hGuQ8kuc9pgc9s8qqaq=dirpe0xb9q8qiLsFr0=vr0=vr0dc8meaabaqaciaacaGaaeqabaqabeGadaaakeaatuuDJXwAKzKCHTgD1jharyqr1ngBPrgigjxyRrxDYbaceaGae8xlHmeaaa@3882@ is compatible with Θ. Hence we only need to show that Θ is compatible with *d*.

Let orderings Θ˜
 MathType@MTEF@5@5@+=feaafiart1ev1aaatCvAUfKttLearuWrP9MDH5MBPbIqV92AaeXatLxBI9gBaebbnrfifHhDYfgasaacH8akY=wiFfYdH8Gipec8Eeeu0xXdbba9frFj0=OqFfea0dXdd9vqai=hGuQ8kuc9pgc9s8qqaq=dirpe0xb9q8qiLsFr0=vr0=vr0dc8meaabaqaciaacaGaaeqabaqabeGadaaakeaacuqHyoqugaacaaaa@2E32@ = *y*_1_, ..., *y*_*n *_of *Y *and Θ˜′
 MathType@MTEF@5@5@+=feaafiart1ev1aaatCvAUfKttLearuWrP9MDH5MBPbIqV92AaeXatLxBI9gBaebbnrfifHhDYfgasaacH8akY=wiFfYdH8Gipec8Eeeu0xXdbba9frFj0=OqFfea0dXdd9vqai=hGuQ8kuc9pgc9s8qqaq=dirpe0xb9q8qiLsFr0=vr0=vr0dc8meaabaqaciaacaGaaeqabaqabeGadaaakeaacuqHyoqugaacgaqbaaaa@2E3D@ = *z*_1_, ..., *z*_*n*-1 _of *Y*' be as in the proof of Proposition 4.3 and let *ω *be the split weight function such that *d *= *d*_*ω*_. Then Θ˜
 MathType@MTEF@5@5@+=feaafiart1ev1aaatCvAUfKttLearuWrP9MDH5MBPbIqV92AaeXatLxBI9gBaebbnrfifHhDYfgasaacH8akY=wiFfYdH8Gipec8Eeeu0xXdbba9frFj0=OqFfea0dXdd9vqai=hGuQ8kuc9pgc9s8qqaq=dirpe0xb9q8qiLsFr0=vr0=vr0dc8meaabaqaciaacaGaaeqabaqabeGadaaakeaacuqHyoqugaacaaaa@2E32@ is compatible with all splits *S *such that *ω*(*S*) > 0. Now consider some split *S *= {*A*, *B*} such that *ω*(*S*) > 0 and assume that *y*_*n *_∈ *B*. Then there exists *i*, *j *∈ {1, ..., *n *- 1}, *i *≤ *j*, such that *A *= {*y*_*i*_, ..., *y*_*j*_}. Note also that, since the distance function *d*' is  compatible with ordering Θ˜′
 MathType@MTEF@5@5@+=feaafiart1ev1aaatCvAUfKttLearuWrP9MDH5MBPbIqV92AaeXatLxBI9gBaebbnrfifHhDYfgasaacH8akY=wiFfYdH8Gipec8Eeeu0xXdbba9frFj0=OqFfea0dXdd9vqai=hGuQ8kuc9pgc9s8qqaq=dirpe0xb9q8qiLsFr0=vr0=vr0dc8meaabaqaciaacaGaaeqabaqabeGadaaakeaacuqHyoqugaacgaqbaaaa@2E3D@ = *z*_1_, ..., *z*_*n*-1 _of *Y*' and, hence, is circular, there exists a unique circular split  weight function *ω*': Ϭ(*Y*') → ℝ_≥0 _with the property that *d*' = *d*_*ω*'_. We divide the remaining argument into five cases.

*Case 1*: *j *≤ 3. Then, clearly, *S *is compatible with Θ.

*Case 2*: *j *≥ 4 and *i *= 1. Define *A*' = {*z*_1_, ..., *z*_*j*-1_} and the split *S*' = {*A*', *Y*'\*A*'} of *Y*'. Then we can express *ω*'(*S*') in terms of *d*' as follows (cf. [12]):

2ω′(S′)=d′(z1,zj)+d′(zj−1,zn−1)−d′(z1,zj−1)−d′(zj,zn−1)=(α+β)d(y1,yj+1)+γd(y2,yj+1)+d(yj,yn)−(α+β)d(y1,yj)−γd(y2,yj)−d(yj+1,yn)≥(α+β+γ)(d(y1,yj+1)+d(yj,yn)−d(y1,yj)−d(yj+1,yn))=2ω(S)
 MathType@MTEF@5@5@+=feaafiart1ev1aaatCvAUfKttLearuWrP9MDH5MBPbIqV92AaeXatLxBI9gBaebbnrfifHhDYfgasaacH8akY=wiFfYdH8Gipec8Eeeu0xXdbba9frFj0=OqFfea0dXdd9vqai=hGuQ8kuc9pgc9s8qqaq=dirpe0xb9q8qiLsFr0=vr0=vr0dc8meaabaqaciaacaGaaeqabaqabeGadaaakeaafaqadeqbbaaaaeaacqaIYaGmiiGacuWFjpWDgaqbaiabcIcaOiqbdofatzaafaGaeiykaKIaeyypa0JafmizaqMbauaacqGGOaakcqWG6bGEdaWgaaWcbaGaeGymaedabeaakiabcYcaSiabdQha6naaBaaaleaacqWGQbGAaeqaaOGaeiykaKIaey4kaSIafmizaqMbauaacqGGOaakcqWG6bGEdaWgaaWcbaGaemOAaOMaeyOeI0IaeGymaedabeaakiabcYcaSiabdQha6naaBaaaleaacqWGUbGBcqGHsislcqaIXaqmaeqaaOGaeiykaKIaeyOeI0IafmizaqMbauaacqGGOaakcqWG6bGEdaWgaaWcbaGaeGymaedabeaakiabcYcaSiabdQha6naaBaaaleaacqWGQbGAcqGHsislcqaIXaqmaeqaaOGaeiykaKIaeyOeI0IafmizaqMbauaacqGGOaakcqWG6bGEdaWgaaWcbaGaemOAaOgabeaakiabcYcaSiabdQha6naaBaaaleaacqWGUbGBcqGHsislcqaIXaqmaeqaaOGaeiykaKcabaGaeyypa0JaeiikaGIae8xSdeMaey4kaSIae8NSdiMaeiykaKIaemizaqMaeiikaGIaemyEaK3aaSbaaSqaaiabigdaXaqabaGccqGGSaalcqWG5bqEdaWgaaWcbaGaemOAaOMaey4kaSIaeGymaedabeaakiabcMcaPiabgUcaRiab=n7aNjabdsgaKjabcIcaOiabdMha5naaBaaaleaacqaIYaGmaeqaaOGaeiilaWIaemyEaK3aaSbaaSqaaiabdQgaQjabgUcaRiabigdaXaqabaGccqGGPaqkcqGHRaWkcqWGKbazcqGGOaakcqWG5bqEdaWgaaWcbaGaemOAaOgabeaakiabcYcaSiabdMha5naaBaaaleaacqWGUbGBaeqaaOGaeiykaKcabiqaaivacaWLjaGaeyOeI0IaeiikaGIae8xSdeMaey4kaSIae8NSdiMaeiykaKIaemizaqMaeiikaGIaemyEaK3aaSbaaSqaaiabigdaXaqabaGccqGGSaalcqWG5bqEdaWgaaWcbaGaemOAaOgabeaakiabcMcaPiabgkHiTiab=n7aNjabdsgaKjabcIcaOiabdMha5naaBaaaleaacqaIYaGmaeqaaOGaeiilaWIaemyEaK3aaSbaaSqaaiabdQgaQbqabaGccqGGPaqkcqGHsislcqWGKbazcqGGOaakcqWG5bqEdaWgaaWcbaGaemOAaOMaey4kaSIaeGymaedabeaakiabcYcaSiabdMha5naaBaaaleaacqWGUbGBaeqaaOGaeiykaKcabaGaeyyzImRaeiikaGIae8xSdeMaey4kaSIae8NSdiMaey4kaSIae83SdCMaeiykaKIaeiikaGIaemizaqMaeiikaGIaemyEaK3aaSbaaSqaaiabigdaXaqabaGccqGGSaalcqWG5bqEdaWgaaWcbaGaemOAaOMaey4kaSIaeGymaedabeaakiabcMcaPiabgUcaRiabdsgaKjabcIcaOiabdMha5naaBaaaleaacqWGQbGAaeqaaOGaeiilaWIaemyEaK3aaSbaaSqaaiabd6gaUbqabaGccqGGPaqkcqGHsislcqWGKbazcqGGOaakcqWG5bqEdaWgaaWcbaGaeGymaedabeaakiabcYcaSiabdMha5naaBaaaleaacqWGQbGAaeqaaOGaeiykaKIaeyOeI0IaemizaqMaeiikaGIaemyEaK3aaSbaaSqaaiabdQgaQjabgUcaRiabigdaXaqabaGccqGGSaalcqWG5bqEdaWgaaWcbaGaemOBa4gabeaakiabcMcaPiabcMcaPaqaaiabg2da9iabikdaYiab=L8a3jabcIcaOiabdofatjabcMcaPaaaaaa@FA52@

Thus, *ω*'(*S*') > 0. Hence, the split *S*' is compatible with the ordering Θ' of *Y*'. But then the split *S *is compatible with the ordering Θ of *Y*.

*Case 3*: *j *≥ 4 and 2 ≤ *i *≤ 3. We only consider the situation when *i *= 2; the situation *i *= 3 is completely analogous. Define *A*' = {*z*_2_, ..., *z*_*j*-1_} and the split *S*' = {*A*', *Y*'\*A*'} of *Y*'. With a similar calculation as made for Case 2 we obtain *ω*'(*S*') ≥ (*α *+ *β*)*ω*(*S*). Hence, *ω*'(*S*') > 0 and, thus, *S*' is compatible with Θ'. But then *S *is compatible with Θ.

*Case 4*: *j *≥ 4 and *i *= 4. This case is similar to Case 2. Define *A*' = {*z*_4_, ..., *z*_*j*-1_} and *S*' = {*A*', *Y*'\*A*'}. We obtain *ω*'(*S*') ≥ *ω*(*S*). Hence, as for Case 2, *ω*'(*S*') > 0 and, thus, *S *is compatible with Θ.

*Case 5*: *j *≥ *i *≥ 5. Define the split *S*' = {*A*, *Y*'\*A*}. Then we have *ω*'(*S*') = *ω*'(*S*') > 0. Hence, *S*' is compatible with Θ' and, thus, *S *is compatible with Θ.   ■

### 4.2 Case 2: The selection case

Now suppose that there are no clusters *C *∈ ℭ
 MathType@MTEF@5@5@+=feaafiart1ev1aaatCvAUfKttLearuWrP9MDH5MBPbIqV92AaeXatLxBI9gBaebbnrfifHhDYfgasaacH8akY=wiFfYdH8Gipec8Eeeu0xXdbba9frFj0=OqFfea0dXdd9vqai=hGuQ8kuc9pgc9s8qqaq=dirpe0xb9q8qiLsFr0=vr0=vr0dc8meaabaqaciaacaGaaeqabaqabeGadaaakeaatuuDJXwAKzKCHTgD1jharyqr1ngBPrgigjxyRrxDYbaceaGae8xlHmeaaa@3882@ with |*C*| ≥ 3. This is the *selection case *in the description of the algorithm.

In line 17 the algorithm selects two clusters that minimize (3):

Q(C1,C2)=(m−2)d¯(C1,C2)−∑C∈ℭ\{C1}d¯(C1,C)−∑C∈ℭ\{C2}d¯(C2,C),
 MathType@MTEF@5@5@+=feaafiart1ev1aaatCvAUfKttLearuWrP9MDH5MBPbIqV92AaeXatLxBI9gBaebbnrfifHhDYfgasaacH8akY=wiFfYdH8Gipec8Eeeu0xXdbba9frFj0=OqFfea0dXdd9vqai=hGuQ8kuc9pgc9s8qqaq=dirpe0xb9q8qiLsFr0=vr0=vr0dc8meaabaqaciaacaGaaeqabaqabeGadaaakeaacqWGrbqucqGGOaakcqWGdbWqdaWgaaWcbaGaeGymaedabeaakiabcYcaSiabdoeadnaaBaaaleaacqaIYaGmaeqaaOGaeiykaKIaeyypa0JaeiikaGIaemyBa0MaeyOeI0IaeGOmaiJaeiykaKIafmizaqMbaebacqGGOaakcqWGdbWqdaWgaaWcbaGaeGymaedabeaakiabcYcaSiabdoeadnaaBaaaleaacqaIYaGmaeqaaOGaeiykaKIaeyOeI0YaaabuaeaacuWGKbazgaqeaiabcIcaOiabdoeadnaaBaaaleaacqaIXaqmaeqaaOGaeiilaWIaem4qamKaeiykaKIaeyOeI0YaaabuaeaacuWGKbazgaqeaiabcIcaOiabdoeadnaaBaaaleaacqaIYaGmaeqaaOGaeiilaWIaem4qamKaeiykaKcaleaacqWGdbWqcqGHiiIZtuuDJXwAKzKCHTgD1jharyqr1ngBPrgigjxyRrxDYbaceaGae8xlHmKaeiixaWLaei4EaSNaem4qam0aaSbaaWqaaiabikdaYaqabaWccqGG9bqFaeqaniabggHiLdaaleaacqWGdbWqcqGHiiIZcqWFTeYqcqGGCbaxcqGG7bWEcqWGdbWqdaWgaaadbaGaeGymaedabeaaliabc2ha9bqab0GaeyyeIuoakiabcYcaSaaa@77C2@

where

d¯(A,B)={0if A=B1|A||B|∑a∈A∑b∈Bd(a,b)if A≠B.
 MathType@MTEF@5@5@+=feaafiart1ev1aaatCvAUfKttLearuWrP9MDH5MBPbIqV92AaeXatLxBI9gBaebbnrfifHhDYfgasaacH8akY=wiFfYdH8Gipec8Eeeu0xXdbba9frFj0=OqFfea0dXdd9vqai=hGuQ8kuc9pgc9s8qqaq=dirpe0xb9q8qiLsFr0=vr0=vr0dc8meaabaqaciaacaGaaeqabaqabeGadaaakeaacuWGKbazgaqeaiabcIcaOiabdgeabjabcYcaSiabdkeacjabcMcaPiabg2da9maaceqabaqbaeaabiGaaaqaaiabicdaWaqaaiabbMgaPjabbAgaMjabbccaGiabdgeabjabg2da9iabdkeacbqaamaalaaabaGaeGymaedabaWaaqWaaeaacqWGbbqqaiaawEa7caGLiWoadaabdaqaaiabdkeacbGaay5bSlaawIa7aaaadaaeqaqaamaaqababaGaemizaqMaeiikaGIaemyyaeMaeiilaWIaemOyaiMaeiykaKcaleaacqWGIbGycqGHiiIZcqWGcbGqaeqaniabggHiLdaaleaacqWGHbqycqGHiiIZcqWGbbqqaeqaniabggHiLdaakeaacqqGPbqAcqqGMbGzcqqGGaaicqWGbbqqcqGHGjsUcqWGcbGqcqGGUaGlaaaacaGL7baaaaa@6003@

Note that d¯
 MathType@MTEF@5@5@+=feaafiart1ev1aaatCvAUfKttLearuWrP9MDH5MBPbIqV92AaeXatLxBI9gBaebbnrfifHhDYfgasaacH8akY=wiFfYdH8Gipec8Eeeu0xXdbba9frFj0=OqFfea0dXdd9vqai=hGuQ8kuc9pgc9s8qqaq=dirpe0xb9q8qiLsFr0=vr0=vr0dc8meaabaqaciaacaGaaeqabaqabeGadaaakeaacuWGKbazgaqeaaaa@2E15@ is a distance function defined on the set of clusters ℭ
 MathType@MTEF@5@5@+=feaafiart1ev1aaatCvAUfKttLearuWrP9MDH5MBPbIqV92AaeXatLxBI9gBaebbnrfifHhDYfgasaacH8akY=wiFfYdH8Gipec8Eeeu0xXdbba9frFj0=OqFfea0dXdd9vqai=hGuQ8kuc9pgc9s8qqaq=dirpe0xb9q8qiLsFr0=vr0=vr0dc8meaabaqaciaacaGaaeqabaqabeGadaaakeaatuuDJXwAKzKCHTgD1jharyqr1ngBPrgigjxyRrxDYbaceaGae8xlHmeaaa@3882@. We will first show that d¯
 MathType@MTEF@5@5@+=feaafiart1ev1aaatCvAUfKttLearuWrP9MDH5MBPbIqV92AaeXatLxBI9gBaebbnrfifHhDYfgasaacH8akY=wiFfYdH8Gipec8Eeeu0xXdbba9frFj0=OqFfea0dXdd9vqai=hGuQ8kuc9pgc9s8qqaq=dirpe0xb9q8qiLsFr0=vr0=vr0dc8meaabaqaciaacaGaaeqabaqabeGadaaakeaacuWGKbazgaqeaaaa@2E15@ is circular. We do this in two steps: Proposition 4.5 and Proposition 4.6.

**Proposition 4.5 **Let *d*: *M *× *M *→ ℝ_≥0 _be a circular distance function and Θ = *x*_1_, ..., *x*_*n *_be an ordering of *M *that is compatible with *d*. Let *M*' = (*M*\{*x*_1_, *x*_2_}) ∪ {*y*} where *y *is a new element not contained in *M*. Define a distance function *d*': *M*' × *M*' → ℝ_≥0 _as follows:

d′(a,b)=d(a,b)for {a,b}⊆M′\{y}d′(y,a)=λd(x1,a)+(1−λ)d(x2,a)for a∈M′\{y},
 MathType@MTEF@5@5@+=feaafiart1ev1aaatCvAUfKttLearuWrP9MDH5MBPbIqV92AaeXatLxBI9gBaebbnrfifHhDYfgasaacH8akY=wiFfYdH8Gipec8Eeeu0xXdbba9frFj0=OqFfea0dXdd9vqai=hGuQ8kuc9pgc9s8qqaq=dirpe0xb9q8qiLsFr0=vr0=vr0dc8meaabaqaciaacaGaaeqabaqabeGadaaakeaafaqaaeGacaaabaGafmizaqMbauaacqGGOaakcqWGHbqycqGGSaalcqWGIbGycqGGPaqkcqGH9aqpcqWGKbazcqGGOaakcqWGHbqycqGGSaalcqWGIbGycqGGPaqkaeaacqqGMbGzcqqGVbWBcqqGYbGCcqqGGaaicqGG7bWEcqWGHbqycqGGSaalcqWGIbGycqGG9bqFcqGHgksZcuWGnbqtgaqbaiabcYfaCjabcUha7jabdMha5jabc2ha9bqaaiqbdsgaKzaafaGaeiikaGIaemyEaKNaeiilaWIaemyyaeMaeiykaKIaeyypa0dcciGae83UdWMaemizaqMaeiikaGIaemiEaG3aaSbaaSqaaiabigdaXaqabaGccqGGSaalcqWGHbqycqGGPaqkcqGHRaWkcqGGOaakcqaIXaqmcqGHsislcqWF7oaBcqGGPaqkcqWGKbazcqGGOaakcqWG4baEdaWgaaWcbaGaeGOmaidabeaakiabcYcaSiabdggaHjabcMcaPaqaaiabbAgaMjabb+gaVjabbkhaYjabbccaGiabdggaHjabgIGiolqbd2eanzaafaGaeiixaWLaei4EaSNaemyEaKNaeiyFa0NaeiilaWcaaaaa@7DC5@

where *λ *is a real number with the property that 0 <*λ *< 1. Then the following hold:

(i) *d*' is circular and compatible with ordering *y*, *x*_3_, ..., *x*_*n *_of *M*'.

(ii) If *z*_1_, ..., *z*_*n*-1 _is an ordering of *M*' that is compatible with *d*' then at least one of the orderings *x*_1_, *x*_2_, *z*_2_, ..., *z*_*n*-1 _or *x*_2_, *x*_1_, *z*_2_, ..., *z*_*n*-1 _of *M *is compatible with *d*.

*Proof*: (i) and (ii) can be proven using convexity arguments, or in a way analogous to our proof of Propositions 4.3 and 4.4, respectively.   ■

**Proposition 4.6 **The distance function d¯
 MathType@MTEF@5@5@+=feaafiart1ev1aaatCvAUfKttLearuWrP9MDH5MBPbIqV92AaeXatLxBI9gBaebbnrfifHhDYfgasaacH8akY=wiFfYdH8Gipec8Eeeu0xXdbba9frFj0=OqFfea0dXdd9vqai=hGuQ8kuc9pgc9s8qqaq=dirpe0xb9q8qiLsFr0=vr0=vr0dc8meaabaqaciaacaGaaeqabaqabeGadaaakeaacuWGKbazgaqeaaaa@2E15@, defined on the individual clusters in ℭ
 MathType@MTEF@5@5@+=feaafiart1ev1aaatCvAUfKttLearuWrP9MDH5MBPbIqV92AaeXatLxBI9gBaebbnrfifHhDYfgasaacH8akY=wiFfYdH8Gipec8Eeeu0xXdbba9frFj0=OqFfea0dXdd9vqai=hGuQ8kuc9pgc9s8qqaq=dirpe0xb9q8qiLsFr0=vr0=vr0dc8meaabaqaciaacaGaaeqabaqabeGadaaakeaatuuDJXwAKzKCHTgD1jharyqr1ngBPrgigjxyRrxDYbaceaGae8xlHmeaaa@3882@, is a circular distance. Moreover, for every ordering *D*_1_, ..., *D*_*k *_of ℭ
 MathType@MTEF@5@5@+=feaafiart1ev1aaatCvAUfKttLearuWrP9MDH5MBPbIqV92AaeXatLxBI9gBaebbnrfifHhDYfgasaacH8akY=wiFfYdH8Gipec8Eeeu0xXdbba9frFj0=OqFfea0dXdd9vqai=hGuQ8kuc9pgc9s8qqaq=dirpe0xb9q8qiLsFr0=vr0=vr0dc8meaabaqaciaacaGaaeqabaqabeGadaaakeaatuuDJXwAKzKCHTgD1jharyqr1ngBPrgigjxyRrxDYbaceaGae8xlHmeaaa@3882@ that is compatible with d¯
 MathType@MTEF@5@5@+=feaafiart1ev1aaatCvAUfKttLearuWrP9MDH5MBPbIqV92AaeXatLxBI9gBaebbnrfifHhDYfgasaacH8akY=wiFfYdH8Gipec8Eeeu0xXdbba9frFj0=OqFfea0dXdd9vqai=hGuQ8kuc9pgc9s8qqaq=dirpe0xb9q8qiLsFr0=vr0=vr0dc8meaabaqaciaacaGaaeqabaqabeGadaaakeaacuWGKbazgaqeaaaa@2E15@ there exist orderings Θ_*i *_of *D*_*i*_, *i *∈ {1, ..., *k*}, such that the ordering Θ_1_, ..., Θ_*k *_of *Y *is compatible with distance function *d*.

*Proof*: We use multiple applications of Proposition 4.5, once for each cluster in ℭ
 MathType@MTEF@5@5@+=feaafiart1ev1aaatCvAUfKttLearuWrP9MDH5MBPbIqV92AaeXatLxBI9gBaebbnrfifHhDYfgasaacH8akY=wiFfYdH8Gipec8Eeeu0xXdbba9frFj0=OqFfea0dXdd9vqai=hGuQ8kuc9pgc9s8qqaq=dirpe0xb9q8qiLsFr0=vr0=vr0dc8meaabaqaciaacaGaaeqabaqabeGadaaakeaatuuDJXwAKzKCHTgD1jharyqr1ngBPrgigjxyRrxDYbaceaGae8xlHmeaaa@3882@ with two elements, and with *λ *= 12
 MathType@MTEF@5@5@+=feaafiart1ev1aaatCvAUfKttLearuWrP9MDH5MBPbIqV92AaeXatLxBI9gBaebbnrfifHhDYfgasaacH8akY=wiFfYdH8Gipec8Eeeu0xXdbba9frFj0=OqFfea0dXdd9vqai=hGuQ8kuc9pgc9s8qqaq=dirpe0xb9q8qiLsFr0=vr0=vr0dc8meaabaqaciaacaGaaeqabaqabeGadaaakeaadaWcaaqaaiabigdaXaqaaiabikdaYaaaaaa@2E9E@ in each case.   ■

We now have the more difficult task of showing that clusters *C*_1 _and *C*_2 _selected by the *Q*-criterion, that is by minimizing (3), are adjacent in at least one ordering of the clusters that is compatible with d¯
 MathType@MTEF@5@5@+=feaafiart1ev1aaatCvAUfKttLearuWrP9MDH5MBPbIqV92AaeXatLxBI9gBaebbnrfifHhDYfgasaacH8akY=wiFfYdH8Gipec8Eeeu0xXdbba9frFj0=OqFfea0dXdd9vqai=hGuQ8kuc9pgc9s8qqaq=dirpe0xb9q8qiLsFr0=vr0=vr0dc8meaabaqaciaacaGaaeqabaqabeGadaaakeaacuWGKbazgaqeaaaa@2E15@, as described in Proposition 4.6. This is the most technical part of the proof. The key step is the inequality established in Lemma 4.7. This is used to prove Theorem 4.8, which establishes that the *Q*-criterion when applied to a circular distance will always select a pair of elements that are adjacent in at least one ordering compatible with the circular distance. As a corollary it will follow that there exists an ordering of the clusters in ℭ
 MathType@MTEF@5@5@+=feaafiart1ev1aaatCvAUfKttLearuWrP9MDH5MBPbIqV92AaeXatLxBI9gBaebbnrfifHhDYfgasaacH8akY=wiFfYdH8Gipec8Eeeu0xXdbba9frFj0=OqFfea0dXdd9vqai=hGuQ8kuc9pgc9s8qqaq=dirpe0xb9q8qiLsFr0=vr0=vr0dc8meaabaqaciaacaGaaeqabaqabeGadaaakeaatuuDJXwAKzKCHTgD1jharyqr1ngBPrgigjxyRrxDYbaceaGae8xlHmeaaa@3882@ compatible with d¯
 MathType@MTEF@5@5@+=feaafiart1ev1aaatCvAUfKttLearuWrP9MDH5MBPbIqV92AaeXatLxBI9gBaebbnrfifHhDYfgasaacH8akY=wiFfYdH8Gipec8Eeeu0xXdbba9frFj0=OqFfea0dXdd9vqai=hGuQ8kuc9pgc9s8qqaq=dirpe0xb9q8qiLsFr0=vr0=vr0dc8meaabaqaciaacaGaaeqabaqabeGadaaakeaacuWGKbazgaqeaaaa@2E15@ where *C*_1 _and *C*_2 _are adjacent.

**Lemma 4.7 **Let Θ = *x*_1_, *x*_2_, ..., *x*_*n *_be an ordering of *M *that is compatible with circular distance *d *on *M *and suppose that 3 ≤ *r *≤ ⌈*n*/2⌉. Let *S *= {*A*, *M*\*A*} be a split compatible with Θ where *A *= {*x*_*i*_, ..., *x*_*j*_}. Define *Q*_*S*_: *M *× *M *→ ℝ by

QS(xi,xj)=(n−2)dS(xi,xj)−∑k=1ndS(xi,xk)−∑k=1ndS(xj,xk)
 MathType@MTEF@5@5@+=feaafiart1ev1aaatCvAUfKttLearuWrP9MDH5MBPbIqV92AaeXatLxBI9gBaebbnrfifHhDYfgasaacH8akY=wiFfYdH8Gipec8Eeeu0xXdbba9frFj0=OqFfea0dXdd9vqai=hGuQ8kuc9pgc9s8qqaq=dirpe0xb9q8qiLsFr0=vr0=vr0dc8meaabaqaciaacaGaaeqabaqabeGadaaakeaacqWGrbqudaWgaaWcbaGaem4uamfabeaakiabcIcaOiabdIha4naaBaaaleaacqWGPbqAaeqaaOGaeiilaWIaemiEaG3aaSbaaSqaaiabdQgaQbqabaGccqGGPaqkcqGH9aqpcqGGOaakcqWGUbGBcqGHsislcqaIYaGmcqGGPaqkcqWGKbazdaWgaaWcbaGaem4uamfabeaakiabcIcaOiabdIha4naaBaaaleaacqWGPbqAaeqaaOGaeiilaWIaemiEaG3aaSbaaSqaaiabdQgaQbqabaGccqGGPaqkcqGHsisldaaeWbqaaiabdsgaKnaaBaaaleaacqWGtbWuaeqaaOGaeiikaGIaemiEaG3aaSbaaSqaaiabdMgaPbqabaGccqGGSaalcqWG4baEdaWgaaWcbaGaem4AaSgabeaakiabcMcaPaWcbaGaem4AaSMaeyypa0JaeGymaedabaGaemOBa4ganiabggHiLdGccqGHsisldaaeWbqaaiabdsgaKnaaBaaaleaacqWGtbWuaeqaaOGaeiikaGIaemiEaG3aaSbaaSqaaiabdQgaQbqabaGccqGGSaalcqWG4baEdaWgaaWcbaGaem4AaSgabeaakiabcMcaPaWcbaGaem4AaSMaeyypa0JaeGymaedabaGaemOBa4ganiabggHiLdaaaa@6FDC@

and let

λ(S)=∑l=1r−1QS(xl,xl+1)−(r−1)QS(x1,xr).
 MathType@MTEF@5@5@+=feaafiart1ev1aaatCvAUfKttLearuWrP9MDH5MBPbIqV92AaeXatLxBI9gBaebbnrfifHhDYfgasaacH8akY=wiFfYdH8Gipec8Eeeu0xXdbba9frFj0=OqFfea0dXdd9vqai=hGuQ8kuc9pgc9s8qqaq=dirpe0xb9q8qiLsFr0=vr0=vr0dc8meaabaqaciaacaGaaeqabaqabeGadaaakeaaiiGacqWF7oaBcqGGOaakcqWGtbWucqGGPaqkcqGH9aqpdaaeWbqaaiabdgfarnaaBaaaleaacqWGtbWuaeqaaOGaeiikaGIaemiEaG3aaSbaaSqaaiabdYgaSbqabaGccqGGSaalcqWG4baEdaWgaaWcbaGaemiBaWMaey4kaSIaeGymaedabeaakiabcMcaPiabgkHiTiabcIcaOiabdkhaYjabgkHiTiabigdaXiabcMcaPiabdgfarnaaBaaaleaacqWGtbWuaeqaaOGaeiikaGIaemiEaG3aaSbaaSqaaiabigdaXaqabaGccqGGSaalcqWG4baEdaWgaaWcbaGaemOCaihabeaakiabcMcaPaWcbaGaemiBaWMaeyypa0JaeGymaedabaGaemOCaiNaeyOeI0IaeGymaedaniabggHiLdGccqGGUaGlaaa@59F9@

(i) If min{|*A*|, |*M*\*A*|} > 1 and |*A *∩ {*x*_1_, *x*_*r*_}| = 1 then *λ*(*S*) < 0.

(ii) Any other split *S *compatible with Θ satisfies *λ*(*S*) ≤ 0.

*Proof*: Expanding *λ*(*S*) gives

λ(S)=(n−2)∑l=1r−1dS(xl,xl+1)−(r−1)(n−2)dS(x1,xr)+(r−2)∑i=1ndS(x1,xl)−2∑l=2r−1∑k=1ndS(xl,xk)+(r−2)∑l=1ndS(xr,xl).
 MathType@MTEF@5@5@+=feaafiart1ev1aaatCvAUfKttLearuWrP9MDH5MBPbIqV92AaeXatLxBI9gBaebbnrfifHhDYfgasaacH8akY=wiFfYdH8Gipec8Eeeu0xXdbba9frFj0=OqFfea0dXdd9vqai=hGuQ8kuc9pgc9s8qqaq=dirpe0xb9q8qiLsFr0=vr0=vr0dc8meaabaqaciaacaGaaeqabaqabeGadaaakeGabaaGeuaabaqadiaaaeaaiiGacqWF7oaBcqGGOaakcqWGtbWucqGGPaqkcqGH9aqpcqGGOaakcqWGUbGBcqGHsislcqaIYaGmcqGGPaqkdaaeWbqaaiabdsgaKnaaBaaaleaacqWGtbWuaeqaaOGaeiikaGIaemiEaG3aaSbaaSqaaiabdYgaSbqabaGccqGGSaalcqWG4baEdaWgaaWcbaGaemiBaWMaey4kaSIaeGymaedabeaakiabcMcaPaWcbaGaemiBaWMaeyypa0JaeGymaedabaGaemOCaiNaeyOeI0IaeGymaedaniabggHiLdaakeaacqGHsislcqGGOaakcqWGYbGCcqGHsislcqaIXaqmcqGGPaqkcqGGOaakcqWGUbGBcqGHsislcqaIYaGmcqGGPaqkcqWGKbazdaWgaaWcbaGaem4uamfabeaakiabcIcaOiabdIha4naaBaaaleaacqaIXaqmaeqaaOGaeiilaWIaemiEaG3aaSbaaSqaaiabdkhaYbqabaGccqGGPaqkaeGabaaCciaaxMaacqGHRaWkcqGGOaakcqWGYbGCcqGHsislcqaIYaGmcqGGPaqkdaaeWbqaaiabdsgaKnaaBaaaleaacqWGtbWuaeqaaOGaeiikaGIaemiEaG3aaSbaaSqaaiabigdaXaqabaGccqGGSaalcqWG4baEdaWgaaWcbaGaemiBaWgabeaakiabcMcaPaWcbaGaemyAaKMaeyypa0JaeGymaedabaGaemOBa4ganiabggHiLdaakeaacqGHsislcqaIYaGmdaaeWbqaamaaqahabaGaemizaq2aaSbaaSqaaiabdofatbqabaGccqGGOaakcqWG4baEdaWgaaWcbaGaemiBaWgabeaakiabcYcaSiabdIha4naaBaaaleaacqWGRbWAaeqaaOGaeiykaKcaleaacqWGRbWAcqGH9aqpcqaIXaqmaeaacqWGUbGBa0GaeyyeIuoaaSqaaiabdYgaSjabg2da9iabikdaYaqaaiabdkhaYjabgkHiTiabigdaXaqdcqGHris5aaGcbiqaaqMacaWLjaGaey4kaSIaeiikaGIaemOCaiNaeyOeI0IaeGOmaiJaeiykaKYaaabCaeaacqWGKbazdaWgaaWcbaGaem4uamfabeaakiabcIcaOiabdIha4naaBaaaleaacqWGYbGCaeqaaOGaeiilaWIaemiEaG3aaSbaaSqaaiabdYgaSbqabaGccqGGPaqkaSqaaiabdYgaSjabg2da9iabigdaXaqaaiabd6gaUbqdcqGHris5aOGaeiOla4cabaaaaaaa@B451@

We divide the rest of our argument into five cases which are summarized in Table 1. For these cases straight-forward calculations yield the entries of Table 2. Using Table 2 we compute *λ*(*S*) in each case.

**Table 1:** List of cases in the proof of Lemma 4.7

Case	*i*	*j*	Case	*i*	*j*
(i)	*i *= 1	1 ≤ *j *<*r*	(iv)	1 <*i *≤ *r*	*r *≤ *j *<*n*
(ii)	*i *= 1	*r *≤ *j *<*n*	(v)	*r *<*i *<*n*	*i *≤ *j *<*n*
(iii)	1 <*i *<*r*	*i *≤ *j *<*r*			

**Table 2:** Precomputed expressions used in the proof of Lemma 4.7

Case	∑l=1r−1dS(xl,xl+1) MathType@MTEF@5@5@+=feaafiart1ev1aaatCvAUfKttLearuWrP9MDH5MBPbIqV92AaeXatLxBI9gBaebbnrfifHhDYfgasaacH8akY=wiFfYdH8Gipec8Eeeu0xXdbba9frFj0=OqFfea0dXdd9vqai=hGuQ8kuc9pgc9s8qqaq=dirpe0xb9q8qiLsFr0=vr0=vr0dc8meaabaqaciaacaGaaeqabaqabeGadaaakeaadaaeWaqaaiabdsgaKnaaBaaaleaacqWGtbWuaeqaaOGaeiikaGIaemiEaG3aaSbaaSqaaiabdYgaSbqabaGccqGGSaalcqWG4baEdaWgaaWcbaGaemiBaWMaey4kaSIaeGymaedabeaakiabcMcaPaWcbaGaemiBaWMaeyypa0JaeGymaedabaGaemOCaiNaeyOeI0IaeGymaedaniabggHiLdaaaa@4289@	*d*_*S*_(*x*_1_, *x*_*r*_)	∑l=1ndS(x1,xl) MathType@MTEF@5@5@+=feaafiart1ev1aaatCvAUfKttLearuWrP9MDH5MBPbIqV92AaeXatLxBI9gBaebbnrfifHhDYfgasaacH8akY=wiFfYdH8Gipec8Eeeu0xXdbba9frFj0=OqFfea0dXdd9vqai=hGuQ8kuc9pgc9s8qqaq=dirpe0xb9q8qiLsFr0=vr0=vr0dc8meaabaqaciaacaGaaeqabaqabeGadaaakeaadaaeWaqaaiabdsgaKnaaBaaaleaacqWGtbWuaeqaaOGaeiikaGIaemiEaG3aaSbaaSqaaiabigdaXaqabaGccqGGSaalcqWG4baEdaWgaaWcbaGaemiBaWgabeaakiabcMcaPaWcbaGaemiBaWMaeyypa0JaeGymaedabaGaemOBa4ganiabggHiLdaaaa@3E61@
(i)	1	1	*n *- *j*
(ii)	0	0	*n *- *j*
(iii)	2	0	*j *- *i *+ 1
(iv)	1	1	*j *- *i *+ 1
(v)	0	0	*j *- *i *+ 1

Case	∑l=2r−1∑k=1ndS(xl,xk) MathType@MTEF@5@5@+=feaafiart1ev1aaatCvAUfKttLearuWrP9MDH5MBPbIqV92AaeXatLxBI9gBaebbnrfifHhDYfgasaacH8akY=wiFfYdH8Gipec8Eeeu0xXdbba9frFj0=OqFfea0dXdd9vqai=hGuQ8kuc9pgc9s8qqaq=dirpe0xb9q8qiLsFr0=vr0=vr0dc8meaabaqaciaacaGaaeqabaqabeGadaaakeaadaaeWaqaamaaqadabaGaemizaq2aaSbaaSqaaiabdofatbqabaGccqGGOaakcqWG4baEdaWgaaWcbaGaemiBaWgabeaakiabcYcaSiabdIha4naaBaaaleaacqWGRbWAaeqaaOGaeiykaKcaleaacqWGRbWAcqGH9aqpcqaIXaqmaeaacqWGUbGBa0GaeyyeIuoaaSqaaiabdYgaSjabg2da9iabikdaYaqaaiabdkhaYjabgkHiTiabigdaXaqdcqGHris5aaaa@4773@		∑l=1ndS(xr,xl) MathType@MTEF@5@5@+=feaafiart1ev1aaatCvAUfKttLearuWrP9MDH5MBPbIqV92AaeXatLxBI9gBaebbnrfifHhDYfgasaacH8akY=wiFfYdH8Gipec8Eeeu0xXdbba9frFj0=OqFfea0dXdd9vqai=hGuQ8kuc9pgc9s8qqaq=dirpe0xb9q8qiLsFr0=vr0=vr0dc8meaabaqaciaacaGaaeqabaqabeGadaaakeaadaaeWaqaaiabdsgaKnaaBaaaleaacqWGtbWuaeqaaOGaeiikaGIaemiEaG3aaSbaaSqaaiabdkhaYbqabaGccqGGSaalcqWG4baEdaWgaaWcbaGaemiBaWgabeaakiabcMcaPaWcbaGaemiBaWMaeyypa0JaeGymaedabaGaemOBa4ganiabggHiLdaaaa@3EDE@

(i)	(*j *- 1)(*n *- *j*) + (*r *- *j *- 1)*j*		*j*
(ii)	(*r *- 2)(*n *- *j*)		*n *- *j*
(iii)	(*j *- *i *+ 1)(*n *- 2*j *+ 2*i *+ *r *- 4)		*j *- *i *+ 1
(iv)	(*i *- 2)(*j *- *i *+ 1) + (*r *- *i*)(*i *- 1 + *n *- *j*)		*i *- 1 + *n *- *j*
(v)	(*r *- 2)(*j *- *i *+ 1)		*j *- *i *+ 1

*Case *(i): We obtain *λ*(*S*) = 2(*j *- 1)(*j *+ 1 - *r*) + 2(*j *- 1)(*j *+ 1 - *n*). Hence, *λ*(*S*) = 0 if *j *= 1 and *λ*(*S*) < 0 if *j *≥ 2.

*Case *(ii): We obtain *λ*(*S*) = 0.

*Case *(iii): We obtain *λ*(*S*) = (*j *- *i*)(4(*j *- *i*) - 2*n *+ 8). Thus, since *j *- *i *≤ *r *- 3 ≤ (*n *+ 1)/2 - 3, *λ*(*S*) = 0 if *i *= *j *and *λ*(*S*) < 0 if *i *<*j*.

*Case *(iv): We obtain *λ*(*S*) = 2(*i *- *r*)(*n *- 2 - (*j *- *i*)) + 2(2 - *i*)(*j *- *i*). Thus, since *j *- *i *≤ *n *- 3, *λ*(*S*) < 0 if *i *<*r*. If *i *= *r *then *λ*(*S*) = 0 if *j *= *r *and *λ*(*S*) < 0 otherwise.

*Case *(v): We obtain *λ*(*S*) = 0.   ■

**Theorem 4.8 **Let *M *be a set of *n *elements and *d*: *M *× *M *→ ℝ_≥0 _be a circular distance function. Suppose that *x*, *y *minimize

Q(x,y)=(n−2)d(x,y)−∑z∈Md(x,z)−∑z∈Md(y,z).
 MathType@MTEF@5@5@+=feaafiart1ev1aaatCvAUfKttLearuWrP9MDH5MBPbIqV92AaeXatLxBI9gBaebbnrfifHhDYfgasaacH8akY=wiFfYdH8Gipec8Eeeu0xXdbba9frFj0=OqFfea0dXdd9vqai=hGuQ8kuc9pgc9s8qqaq=dirpe0xb9q8qiLsFr0=vr0=vr0dc8meaabaqaciaacaGaaeqabaqabeGadaaakeaacqWGrbqucqGGOaakcqWG4baEcqGGSaalcqWG5bqEcqGGPaqkcqGH9aqpcqGGOaakcqWGUbGBcqGHsislcqaIYaGmcqGGPaqkcqWGKbazcqGGOaakcqWG4baEcqGGSaalcqWG5bqEcqGGPaqkcqGHsisldaaeqbqaaiabdsgaKjabcIcaOiabdIha4jabcYcaSiabdQha6jabcMcaPaWcbaGaemOEaONaeyicI4Saemyta0eabeqdcqGHris5aOGaeyOeI0YaaabuaeaacqWGKbazcqGGOaakcqWG5bqEcqGGSaalcqWG6bGEcqGGPaqkaSqaaiabdQha6jabgIGiolabd2eanbqab0GaeyyeIuoakiabc6caUaaa@5D44@

Then there is an ordering of *M *that is compatible with *d *in which *x *and *y *are adjacent.

*Proof*: Let Θ = *x*_1_, ..., *x*_*n *_be an ordering of *M *that is compatible with *d*. Suppose that *Q*(*x*_1_, *x*_*r*_) ≤ *Q*(*x*, *y*) for all *x*, *y *where, without loss of generality, 2 ≤ *r *≤⌈*n*/2⌉. If *r *= 2 then we are done, so we assume *r *≥ 3. Let *ω *be the (circular) split weight function for which *d *= *d*_*ω*_, so Θ is compatible with *ω*. Let Θ* be the ordering obtained by removing *x*_*r *_from Θ and re-inserting it immediately after *x*_1_. We claim that Θ* is also compatible with *ω*.

As in Lemma 4.7, for any split *S *compatible with Θ we define

λ(S)=∑l=1r−1QS(xl,xl+1)−(r−1)QS(x1,xr).
 MathType@MTEF@5@5@+=feaafiart1ev1aaatCvAUfKttLearuWrP9MDH5MBPbIqV92AaeXatLxBI9gBaebbnrfifHhDYfgasaacH8akY=wiFfYdH8Gipec8Eeeu0xXdbba9frFj0=OqFfea0dXdd9vqai=hGuQ8kuc9pgc9s8qqaq=dirpe0xb9q8qiLsFr0=vr0=vr0dc8meaabaqaciaacaGaaeqabaqabeGadaaakeaaiiGacqWF7oaBcqGGOaakcqWGtbWucqGGPaqkcqGH9aqpdaaeWbqaaiabdgfarnaaBaaaleaacqWGtbWuaeqaaOGaeiikaGIaemiEaG3aaSbaaSqaaiabdYgaSbqabaGccqGGSaalcqWG4baEdaWgaaWcbaGaemiBaWMaey4kaSIaeGymaedabeaakiabcMcaPiabgkHiTiabcIcaOiabdkhaYjabgkHiTiabigdaXiabcMcaPiabdgfarnaaBaaaleaacqWGtbWuaeqaaOGaeiikaGIaemiEaG3aaSbaaSqaaiabigdaXaqabaGccqGGSaalcqWG4baEdaWgaaWcbaGaemOCaihabeaakiabcMcaPiabc6caUaWcbaGaemiBaWMaeyypa0JaeGymaedabaGaemOCaiNaeyOeI0IaeGymaedaniabggHiLdaaaa@59EF@

By the choice of *x*_1 _and *x*_*r *_we have

(r−1)Q(x1,xr)≤∑l=1r−1Q(xl,xl+1).
 MathType@MTEF@5@5@+=feaafiart1ev1aaatCvAUfKttLearuWrP9MDH5MBPbIqV92AaeXatLxBI9gBaebbnrfifHhDYfgasaacH8akY=wiFfYdH8Gipec8Eeeu0xXdbba9frFj0=OqFfea0dXdd9vqai=hGuQ8kuc9pgc9s8qqaq=dirpe0xb9q8qiLsFr0=vr0=vr0dc8meaabaqaciaacaGaaeqabaqabeGadaaakeaacqGGOaakcqWGYbGCcqGHsislcqaIXaqmcqGGPaqkcqWGrbqucqGGOaakcqWG4baEdaWgaaWcbaGaeGymaedabeaakiabcYcaSiabdIha4naaBaaaleaacqWGYbGCaeqaaOGaeiykaKIaeyizIm6aaabCaeaacqWGrbqucqGGOaakcqWG4baEdaWgaaWcbaGaemiBaWgabeaakiabcYcaSiabdIha4naaBaaaleaacqWGSbaBcqGHRaWkcqaIXaqmaeqaaOGaeiykaKcaleaacqWGSbaBcqGH9aqpcqaIXaqmaeaacqWGYbGCcqGHsislcqaIXaqma0GaeyyeIuoakiabc6caUaaa@5255@

Since *Q *is linear, and *d *= Σ_*S*∈Ϭ(*X*)_*ω*(*S*)*d*_*S *_by Lemma 4.7 we have

0≤∑l=1r−1Q(xl,xl+1)−(r−1)Q(x1,xr)=∑Sω(S)(∑l=1r−1QS(xl,xl+1)−(r−1)QS(x1,xr))=∑Sω(S)λ(S)≤0.
 MathType@MTEF@5@5@+=feaafiart1ev1aaatCvAUfKttLearuWrP9MDH5MBPbIqV92AaeXatLxBI9gBaebbnrfifHhDYfgasaacH8akY=wiFfYdH8Gipec8Eeeu0xXdbba9frFj0=OqFfea0dXdd9vqai=hGuQ8kuc9pgc9s8qqaq=dirpe0xb9q8qiLsFr0=vr0=vr0dc8meaabaqaciaacaGaaeqabaqabeGadaaakeaafaqadeWabaaabaGaeGimaaJaeyizIm6aaabCaeaacqWGrbqucqGGOaakcqWG4baEdaWgaaWcbaGaemiBaWgabeaakiabcYcaSiabdIha4naaBaaaleaacqWGSbaBcqGHRaWkcqaIXaqmaeqaaOGaeiykaKIaeyOeI0IaeiikaGIaemOCaiNaeyOeI0IaeGymaeJaeiykaKIaemyuaeLaeiikaGIaemiEaG3aaSbaaSqaaiabigdaXaqabaGccqGGSaalcqWG4baEdaWgaaWcbaGaemOCaihabeaakiabcMcaPaWcbaGaemiBaWMaeyypa0JaeGymaedabaGaemOCaiNaeyOeI0IaeGymaedaniabggHiLdaakeaacqGH9aqpdaaeqbqaaGGaciab=L8a3jabcIcaOiabdofatjabcMcaPmaabmaabaWaaabCaeaacqWGrbqudaWgaaWcbaGaem4uamfabeaakiabcIcaOiabdIha4naaBaaaleaacqWGSbaBaeqaaOGaeiilaWIaemiEaG3aaSbaaSqaaiabdYgaSjabgUcaRiabigdaXaqabaGccqGGPaqkcqGHsislcqGGOaakcqWGYbGCcqGHsislcqaIXaqmcqGGPaqkcqWGrbqudaWgaaWcbaGaem4uamfabeaakiabcIcaOiabdIha4naaBaaaleaacqaIXaqmaeqaaOGaeiilaWIaemiEaG3aaSbaaSqaaiabdkhaYbqabaGccqGGPaqkaSqaaiabdYgaSjabg2da9iabigdaXaqaaiabdkhaYjabgkHiTiabigdaXaqdcqGHris5aaGccaGLOaGaayzkaaaaleaacqWGtbWuaeqaniabggHiLdaakeaacqGH9aqpdaaeqbqaaiab=L8a3jabcIcaOiabdofatjabcMcaPiab=T7aSjabcIcaOiabdofatjabcMcaPiabgsMiJkabicdaWaWcbaGaem4uamfabeqdcqGHris5aOGaeiOla4caaaaa@95E6@

Now consider any split *S *compatible with Θ but not Θ*. Then *S *satisfies the conditions in Lemma 4.7 (i), giving *λ*(*S*) < 0 and hence *ω*(*S*) = 0. Thus there are no splits in the support of *ω *that are not compatible with Θ*, and Θ* is compatible with *ω *and, hence, *d*. Thus *x*_1 _and *x*_*r *_are adjacent in an ordering Θ* compatible with *d*.   ■

**Corollary 4.9 **Let *C*_1 _and *C*_2 _be the two clusters selected in line 17 of procedure FINDORDERING. Then there exists an ordering Θ* = *D*_1_, ..., *D*_*k *_of ℭ
 MathType@MTEF@5@5@+=feaafiart1ev1aaatCvAUfKttLearuWrP9MDH5MBPbIqV92AaeXatLxBI9gBaebbnrfifHhDYfgasaacH8akY=wiFfYdH8Gipec8Eeeu0xXdbba9frFj0=OqFfea0dXdd9vqai=hGuQ8kuc9pgc9s8qqaq=dirpe0xb9q8qiLsFr0=vr0=vr0dc8meaabaqaciaacaGaaeqabaqabeGadaaakeaatuuDJXwAKzKCHTgD1jharyqr1ngBPrgigjxyRrxDYbaceaGae8xlHmeaaa@3882@ such that *D*_1 _= *C*_1_, *D*_2 _= *C*_2 _and d¯
 MathType@MTEF@5@5@+=feaafiart1ev1aaatCvAUfKttLearuWrP9MDH5MBPbIqV92AaeXatLxBI9gBaebbnrfifHhDYfgasaacH8akY=wiFfYdH8Gipec8Eeeu0xXdbba9frFj0=OqFfea0dXdd9vqai=hGuQ8kuc9pgc9s8qqaq=dirpe0xb9q8qiLsFr0=vr0=vr0dc8meaabaqaciaacaGaaeqabaqabeGadaaakeaacuWGKbazgaqeaaaa@2E15@ is compatible with Θ*.

After selecting *C*_1 _and *C*_2 _the procedure FINDORDERING removes these clusters from the collection and replaces them with their union *C*' = *C*_1 _∪ *C*_2_. It also assigns an ordering Θ_*C*' _to the cluster.

FINDORDERING is then called recursively. The following is directly analogous to Proposition 4.3.

**Proposition 4.10 **There exists an ordering of *Y *that is compatible with collection ℭ′
 MathType@MTEF@5@5@+=feaafiart1ev1aaatCvAUfKttLearuWrP9MDH5MBPbIqV92AaeXatLxBI9gBaebbnrfifHhDYfgasaacH8akY=wiFfYdH8Gipec8Eeeu0xXdbba9frFj0=OqFfea0dXdd9vqai=hGuQ8kuc9pgc9s8qqaq=dirpe0xb9q8qiLsFr0=vr0=vr0dc8meaabaqaciaacaGaaeqabaqabeGadaaakeaatuuDJXwAKzKCHTgD1jharyqr1ngBPrgigjxyRrxDYbaceaGaf8xlHmKbauaaaaa@388E@ and split weight function *ω*.

*Proof*: We already know by Proposition 4.9 and Proposition 4.6 that there exists an ordering Θ˜
 MathType@MTEF@5@5@+=feaafiart1ev1aaatCvAUfKttLearuWrP9MDH5MBPbIqV92AaeXatLxBI9gBaebbnrfifHhDYfgasaacH8akY=wiFfYdH8Gipec8Eeeu0xXdbba9frFj0=OqFfea0dXdd9vqai=hGuQ8kuc9pgc9s8qqaq=dirpe0xb9q8qiLsFr0=vr0=vr0dc8meaabaqaciaacaGaaeqabaqabeGadaaakeaacuqHyoqugaacaaaa@2E32@ = *y*_1_, ..., *y*_*n *_of *Y *that is compatible with ℭ
 MathType@MTEF@5@5@+=feaafiart1ev1aaatCvAUfKttLearuWrP9MDH5MBPbIqV92AaeXatLxBI9gBaebbnrfifHhDYfgasaacH8akY=wiFfYdH8Gipec8Eeeu0xXdbba9frFj0=OqFfea0dXdd9vqai=hGuQ8kuc9pgc9s8qqaq=dirpe0xb9q8qiLsFr0=vr0=vr0dc8meaabaqaciaacaGaaeqabaqabeGadaaakeaatuuDJXwAKzKCHTgD1jharyqr1ngBPrgigjxyRrxDYbaceaGae8xlHmeaaa@3882@ and *ω *and, in addition, also satisfies one of the following properties:

C1={y1} and C2={y2}C1={y1} and C2={y2,y3}C1={y1,y2} and C2={y3}C1={y1,y2} and C2{y3,y4}.
 MathType@MTEF@5@5@+=feaafiart1ev1aaatCvAUfKttLearuWrP9MDH5MBPbIqV92AaeXatLxBI9gBaebbnrfifHhDYfgasaacH8akY=wiFfYdH8Gipec8Eeeu0xXdbba9frFj0=OqFfea0dXdd9vqai=hGuQ8kuc9pgc9s8qqaq=dirpe0xb9q8qiLsFr0=vr0=vr0dc8meaabaqaciaacaGaaeqabaqabeGadaaakeaafaqaaeGacaaabaGaem4qam0aaSbaaSqaaiabigdaXaqabaGccqGH9aqpcqGG7bWEcqWG5bqEdaWgaaWcbaGaeGymaedabeaakiabc2ha9jabbccaGiabbggaHjabb6gaUjabbsgaKjabbccaGiabdoeadnaaBaaaleaacqaIYaGmaeqaaOGaeyypa0Jaei4EaSNaemyEaK3aaSbaaSqaaiabikdaYaqabaGccqGG9bqFaeaacqWGdbWqdaWgaaWcbaGaeGymaedabeaakiabg2da9iabcUha7jabdMha5naaBaaaleaacqaIXaqmaeqaaOGaeiyFa0NaeeiiaaIaeeyyaeMaeeOBa4MaeeizaqMaeeiiaaIaem4qam0aaSbaaSqaaiabikdaYaqabaGccqGH9aqpcqGG7bWEcqWG5bqEdaWgaaWcbaGaeGOmaidabeaakiabcYcaSiabdMha5naaBaaaleaacqaIZaWmaeqaaOGaeiyFa0habaGaem4qam0aaSbaaSqaaiabigdaXaqabaGccqGH9aqpcqGG7bWEcqWG5bqEdaWgaaWcbaGaeGymaedabeaakiabcYcaSiabdMha5naaBaaaleaacqaIYaGmaeqaaOGaeiyFa0NaeeiiaaIaeeyyaeMaeeOBa4MaeeizaqMaeeiiaaIaem4qam0aaSbaaSqaaiabikdaYaqabaGccqGH9aqpcqGG7bWEcqWG5bqEdaWgaaWcbaGaeG4mamdabeaakiabc2ha9bqaaiabdoeadnaaBaaaleaacqaIXaqmaeqaaOGaeyypa0Jaei4EaSNaemyEaK3aaSbaaSqaaiabigdaXaqabaGccqGGSaalcqWG5bqEdaWgaaWcbaGaeGOmaidabeaakiabc2ha9jabbccaGiabbggaHjabb6gaUjabbsgaKjabbccaGiabdoeadnaaBaaaleaacqaIYaGmaeqaaOGaei4EaSNaemyEaK3aaSbaaSqaaiabiodaZaqabaGccqGGSaalcqWG5bqEdaWgaaWcbaGaeGinaqdabeaakiabc2ha9jabc6caUaaaaaa@97C3@

If *x*_1 _∈ *C*_1 _and *x*_2 _∈ *C*_2 _are selected such that Θ˜
 MathType@MTEF@5@5@+=feaafiart1ev1aaatCvAUfKttLearuWrP9MDH5MBPbIqV92AaeXatLxBI9gBaebbnrfifHhDYfgasaacH8akY=wiFfYdH8Gipec8Eeeu0xXdbba9frFj0=OqFfea0dXdd9vqai=hGuQ8kuc9pgc9s8qqaq=dirpe0xb9q8qiLsFr0=vr0=vr0dc8meaabaqaciaacaGaaeqabaqabeGadaaakeaacuqHyoqugaacaaaa@2E32@ is also compatible with ℭ′
 MathType@MTEF@5@5@+=feaafiart1ev1aaatCvAUfKttLearuWrP9MDH5MBPbIqV92AaeXatLxBI9gBaebbnrfifHhDYfgasaacH8akY=wiFfYdH8Gipec8Eeeu0xXdbba9frFj0=OqFfea0dXdd9vqai=hGuQ8kuc9pgc9s8qqaq=dirpe0xb9q8qiLsFr0=vr0=vr0dc8meaabaqaciaacaGaaeqabaqabeGadaaakeaatuuDJXwAKzKCHTgD1jharyqr1ngBPrgigjxyRrxDYbaceaGaf8xlHmKbauaaaaa@388E@ then we are done. Otherwise we have to construct a suitable new ordering Θ˜′
 MathType@MTEF@5@5@+=feaafiart1ev1aaatCvAUfKttLearuWrP9MDH5MBPbIqV92AaeXatLxBI9gBaebbnrfifHhDYfgasaacH8akY=wiFfYdH8Gipec8Eeeu0xXdbba9frFj0=OqFfea0dXdd9vqai=hGuQ8kuc9pgc9s8qqaq=dirpe0xb9q8qiLsFr0=vr0=vr0dc8meaabaqaciaacaGaaeqabaqabeGadaaakeaacuqHyoqugaacgaqbaaaa@2E3D@ of *Y*. There are, up to symmetric situations with roles of *C*_1 _and *C*_2 _swapped, only two cases we need to consider.

*Case 1*: *C*_1 _= {*y*_1_, *y*_2_}, *x*_1 _= *y*_1 _and *x*_2 _= *y*_3_. We want to show that ordering Θ˜′
 MathType@MTEF@5@5@+=feaafiart1ev1aaatCvAUfKttLearuWrP9MDH5MBPbIqV92AaeXatLxBI9gBaebbnrfifHhDYfgasaacH8akY=wiFfYdH8Gipec8Eeeu0xXdbba9frFj0=OqFfea0dXdd9vqai=hGuQ8kuc9pgc9s8qqaq=dirpe0xb9q8qiLsFr0=vr0=vr0dc8meaabaqaciaacaGaaeqabaqabeGadaaakeaacuqHyoqugaacgaqbaaaa@2E3D@ = *y*_2_, *y*_1_, *y*_3_, ..., *y*_*n *_is compatible with *ω*. To this end we first show that Q^
 MathType@MTEF@5@5@+=feaafiart1ev1aaatCvAUfKttLearuWrP9MDH5MBPbIqV92AaeXatLxBI9gBaebbnrfifHhDYfgasaacH8akY=wiFfYdH8Gipec8Eeeu0xXdbba9frFj0=OqFfea0dXdd9vqai=hGuQ8kuc9pgc9s8qqaq=dirpe0xb9q8qiLsFr0=vr0=vr0dc8meaabaqaciaacaGaaeqabaqabeGadaaakeaacuWGrbqugaqcaaaa@2DE7@[*d*](*y*_2_, *y*_3_) ≤ Q^
 MathType@MTEF@5@5@+=feaafiart1ev1aaatCvAUfKttLearuWrP9MDH5MBPbIqV92AaeXatLxBI9gBaebbnrfifHhDYfgasaacH8akY=wiFfYdH8Gipec8Eeeu0xXdbba9frFj0=OqFfea0dXdd9vqai=hGuQ8kuc9pgc9s8qqaq=dirpe0xb9q8qiLsFr0=vr0=vr0dc8meaabaqaciaacaGaaeqabaqabeGadaaakeaacuWGrbqugaqcaaaa@2DE7@[*d*](*y*_1_, *y*_3_). It suffices to establish this inequality for all split metrics *d*_*S *_with *S *∈ SΘ˜
 MathType@MTEF@5@5@+=feaafiart1ev1aaatCvAUfKttLearuWrP9MDH5MBPbIqV92AaeXatLxBI9gBaebbnrfifHhDYfgasaacH8akY=wiFfYdH8Gipec8Eeeu0xXdbba9frFj0=OqFfea0dXdd9vqai=hGuQ8kuc9pgc9s8qqaq=dirpe0xb9q8qiLsFr0=vr0=vr0dc8meaabaqaciaacaGaaeqabaqabeGadaaakeaatuuDJXwAKzKCHTgD1jharyqr1ngBPrgigjxyRrxDYbaceaWccqWFsa=udaWgaaadbaGafuiMdeLbaGaaaeqaaaaa@3B04@. Define the set of splits

Ϭ' = {{{*y*_2_, ..., *y*_*i*_}, *Y*\{*y*_2_, ..., *y*_*i*_}}|3 ≤ *i *≤ *n *- 1}.

By a case analysis similar to the one applied in the proof of Lemma 4.7 we obtain the following:

• Q^
 MathType@MTEF@5@5@+=feaafiart1ev1aaatCvAUfKttLearuWrP9MDH5MBPbIqV92AaeXatLxBI9gBaebbnrfifHhDYfgasaacH8akY=wiFfYdH8Gipec8Eeeu0xXdbba9frFj0=OqFfea0dXdd9vqai=hGuQ8kuc9pgc9s8qqaq=dirpe0xb9q8qiLsFr0=vr0=vr0dc8meaabaqaciaacaGaaeqabaqabeGadaaakeaacuWGrbqugaqcaaaa@2DE7@[*d*_*S*_](*y*_2_, *y*_3_) = Q^
 MathType@MTEF@5@5@+=feaafiart1ev1aaatCvAUfKttLearuWrP9MDH5MBPbIqV92AaeXatLxBI9gBaebbnrfifHhDYfgasaacH8akY=wiFfYdH8Gipec8Eeeu0xXdbba9frFj0=OqFfea0dXdd9vqai=hGuQ8kuc9pgc9s8qqaq=dirpe0xb9q8qiLsFr0=vr0=vr0dc8meaabaqaciaacaGaaeqabaqabeGadaaakeaacuWGrbqugaqcaaaa@2DE7@[*d*_*S*_](*y*_1_, *y*_3_) if *S *∈ SΘ^
 MathType@MTEF@5@5@+=feaafiart1ev1aaatCvAUfKttLearuWrP9MDH5MBPbIqV92AaeXatLxBI9gBaebbnrfifHhDYfgasaacH8akY=wiFfYdH8Gipec8Eeeu0xXdbba9frFj0=OqFfea0dXdd9vqai=hGuQ8kuc9pgc9s8qqaq=dirpe0xb9q8qiLsFr0=vr0=vr0dc8meaabaqaciaacaGaaeqabaqabeGadaaakeaatuuDJXwAKzKCHTgD1jharyqr1ngBPrgigjxyRrxDYbaceaWccqWFsa=udaWgaaadbaGafuiMdeLbaKaaaeqaaaaa@3B05@\Ϭ', and

• Q^
 MathType@MTEF@5@5@+=feaafiart1ev1aaatCvAUfKttLearuWrP9MDH5MBPbIqV92AaeXatLxBI9gBaebbnrfifHhDYfgasaacH8akY=wiFfYdH8Gipec8Eeeu0xXdbba9frFj0=OqFfea0dXdd9vqai=hGuQ8kuc9pgc9s8qqaq=dirpe0xb9q8qiLsFr0=vr0=vr0dc8meaabaqaciaacaGaaeqabaqabeGadaaakeaacuWGrbqugaqcaaaa@2DE7@[*d*_*S*_](*y*_2_, *y*_3_) <Q^
 MathType@MTEF@5@5@+=feaafiart1ev1aaatCvAUfKttLearuWrP9MDH5MBPbIqV92AaeXatLxBI9gBaebbnrfifHhDYfgasaacH8akY=wiFfYdH8Gipec8Eeeu0xXdbba9frFj0=OqFfea0dXdd9vqai=hGuQ8kuc9pgc9s8qqaq=dirpe0xb9q8qiLsFr0=vr0=vr0dc8meaabaqaciaacaGaaeqabaqabeGadaaakeaacuWGrbqugaqcaaaa@2DE7@[*d*_*S*_](*y*_1_, *y*_3_) if *S *∈ Ϭ'.

But then, since Q^
 MathType@MTEF@5@5@+=feaafiart1ev1aaatCvAUfKttLearuWrP9MDH5MBPbIqV92AaeXatLxBI9gBaebbnrfifHhDYfgasaacH8akY=wiFfYdH8Gipec8Eeeu0xXdbba9frFj0=OqFfea0dXdd9vqai=hGuQ8kuc9pgc9s8qqaq=dirpe0xb9q8qiLsFr0=vr0=vr0dc8meaabaqaciaacaGaaeqabaqabeGadaaakeaacuWGrbqugaqcaaaa@2DE7@[*d*](*y*_1_, *y*_3_) is minimum, Q^
 MathType@MTEF@5@5@+=feaafiart1ev1aaatCvAUfKttLearuWrP9MDH5MBPbIqV92AaeXatLxBI9gBaebbnrfifHhDYfgasaacH8akY=wiFfYdH8Gipec8Eeeu0xXdbba9frFj0=OqFfea0dXdd9vqai=hGuQ8kuc9pgc9s8qqaq=dirpe0xb9q8qiLsFr0=vr0=vr0dc8meaabaqaciaacaGaaeqabaqabeGadaaakeaacuWGrbqugaqcaaaa@2DE7@[*d*](*y*_2_, *y*_3_) = Q^
 MathType@MTEF@5@5@+=feaafiart1ev1aaatCvAUfKttLearuWrP9MDH5MBPbIqV92AaeXatLxBI9gBaebbnrfifHhDYfgasaacH8akY=wiFfYdH8Gipec8Eeeu0xXdbba9frFj0=OqFfea0dXdd9vqai=hGuQ8kuc9pgc9s8qqaq=dirpe0xb9q8qiLsFr0=vr0=vr0dc8meaabaqaciaacaGaaeqabaqabeGadaaakeaacuWGrbqugaqcaaaa@2DE7@[*d*](*y*_1_, *y*_3_). Thus, by the above strict inequality, for every split *S *∈ Ϭ' we must have *ω*(*S*) = 0. Hence, *ω *is compatible with Θ˜′
 MathType@MTEF@5@5@+=feaafiart1ev1aaatCvAUfKttLearuWrP9MDH5MBPbIqV92AaeXatLxBI9gBaebbnrfifHhDYfgasaacH8akY=wiFfYdH8Gipec8Eeeu0xXdbba9frFj0=OqFfea0dXdd9vqai=hGuQ8kuc9pgc9s8qqaq=dirpe0xb9q8qiLsFr0=vr0=vr0dc8meaabaqaciaacaGaaeqabaqabeGadaaakeaacuqHyoqugaacgaqbaaaa@2E3D@.

*Case 2*: *C*_1 _= {*y*_1_, *y*_2_}, *C*_2 _= {*y*_3_, *y*_4_}, *x*_1 _= *y*_1_, *x*_2 _= *y*_4 _and *n *≥ 5. We want to show that Θ˜′
 MathType@MTEF@5@5@+=feaafiart1ev1aaatCvAUfKttLearuWrP9MDH5MBPbIqV92AaeXatLxBI9gBaebbnrfifHhDYfgasaacH8akY=wiFfYdH8Gipec8Eeeu0xXdbba9frFj0=OqFfea0dXdd9vqai=hGuQ8kuc9pgc9s8qqaq=dirpe0xb9q8qiLsFr0=vr0=vr0dc8meaabaqaciaacaGaaeqabaqabeGadaaakeaacuqHyoqugaacgaqbaaaa@2E3D@ = *y*_2_, *y*_1_, *y*_4_, *y*_3_, *y*_5_, ..., *y*_*n *_is compatible with *ω*. A similar argument to the one used in Case 1 shows that for every split *S *in

Ϭ' = {{{*y*_2_, ..., *y*_*i*_}, *Y*\{*y*_2_, ..., *y*_*i*_}}|3 ≤ *i *≤ *n *- 1} ∪ {{{*y*_4_, ..., *y*_*i*_}, *Y*\{*y*_2_, ..., *y*_*i*_}}|5 ≤ *i *≤ *n*}

we must have *ω*(*S*) = 0. Thus, *ω *is compatible with Θ˜′
 MathType@MTEF@5@5@+=feaafiart1ev1aaatCvAUfKttLearuWrP9MDH5MBPbIqV92AaeXatLxBI9gBaebbnrfifHhDYfgasaacH8akY=wiFfYdH8Gipec8Eeeu0xXdbba9frFj0=OqFfea0dXdd9vqai=hGuQ8kuc9pgc9s8qqaq=dirpe0xb9q8qiLsFr0=vr0=vr0dc8meaabaqaciaacaGaaeqabaqabeGadaaakeaacuqHyoqugaacgaqbaaaa@2E3D@.   ■

Now, by Proposition 4.10, we can apply the induction hypothesis and conclude that the recursive call FINDORDERING(ℭ′
 MathType@MTEF@5@5@+=feaafiart1ev1aaatCvAUfKttLearuWrP9MDH5MBPbIqV92AaeXatLxBI9gBaebbnrfifHhDYfgasaacH8akY=wiFfYdH8Gipec8Eeeu0xXdbba9frFj0=OqFfea0dXdd9vqai=hGuQ8kuc9pgc9s8qqaq=dirpe0xb9q8qiLsFr0=vr0=vr0dc8meaabaqaciaacaGaaeqabaqabeGadaaakeaatuuDJXwAKzKCHTgD1jharyqr1ngBPrgigjxyRrxDYbaceaGaf8xlHmKbauaaaaa@388E@, *d*) will return an ordering Θ compatible with ℭ′
 MathType@MTEF@5@5@+=feaafiart1ev1aaatCvAUfKttLearuWrP9MDH5MBPbIqV92AaeXatLxBI9gBaebbnrfifHhDYfgasaacH8akY=wiFfYdH8Gipec8Eeeu0xXdbba9frFj0=OqFfea0dXdd9vqai=hGuQ8kuc9pgc9s8qqaq=dirpe0xb9q8qiLsFr0=vr0=vr0dc8meaabaqaciaacaGaaeqabaqabeGadaaakeaatuuDJXwAKzKCHTgD1jharyqr1ngBPrgigjxyRrxDYbaceaGaf8xlHmKbauaaaaa@388E@ and *d*. Since Θ will order *C*' according to Θ_*C*' _(or its reverse), we have that Θ is compatible with *C*_1 _and *C*_2_. Thus Θ is compatible with ℭ
 MathType@MTEF@5@5@+=feaafiart1ev1aaatCvAUfKttLearuWrP9MDH5MBPbIqV92AaeXatLxBI9gBaebbnrfifHhDYfgasaacH8akY=wiFfYdH8Gipec8Eeeu0xXdbba9frFj0=OqFfea0dXdd9vqai=hGuQ8kuc9pgc9s8qqaq=dirpe0xb9q8qiLsFr0=vr0=vr0dc8meaabaqaciaacaGaaeqabaqabeGadaaakeaatuuDJXwAKzKCHTgD1jharyqr1ngBPrgigjxyRrxDYbaceaGae8xlHmeaaa@3882@ and *d*, completing the proof of Theorem 4.2.   □

**Remark 4.11 **Note that we have shown that Corollary 4.9 holds under the assumption that (in view of line 6) every cluster in ℭ
 MathType@MTEF@5@5@+=feaafiart1ev1aaatCvAUfKttLearuWrP9MDH5MBPbIqV92AaeXatLxBI9gBaebbnrfifHhDYfgasaacH8akY=wiFfYdH8Gipec8Eeeu0xXdbba9frFj0=OqFfea0dXdd9vqai=hGuQ8kuc9pgc9s8qqaq=dirpe0xb9q8qiLsFr0=vr0=vr0dc8meaabaqaciaacaGaaeqabaqabeGadaaakeaatuuDJXwAKzKCHTgD1jharyqr1ngBPrgigjxyRrxDYbaceaGae8xlHmeaaa@3882@ contains at most two elements. However, it is possible to prove this result in the more general setting where clusters can have arbitrary size. In principle, this could yield a consistent variation of the Neighbor-Net algorithm that is analogous to the recently introduced QNet algorithm [16], where, instead of reducing the size of clusters when they have more than two elements, the reduction case is skipped entirely and clusters are pairwise combined until only one cluster is left. However, we suspect that such a method would probably not work well in practice since the reduced distances have smaller variance than the original distances.
